# Simulation of Self-Assembled Monolayers of Polyalanine α‑Helices: Development and Application of an Effective Potential for Film Structure Predictions

**DOI:** 10.1021/acsami.6c01087

**Published:** 2026-05-22

**Authors:** Hadis Ghodrati, Kevin Preis, Thi Ngoc Ha Nguyen, Christoph Tegenkamp, Sibylle Gemming, Jeffrey Kelling, Florian Günther

**Affiliations:** † Institute of Physics, 38869 Technische Universität Chemnitz 09107, Chemnitz, Germany; ‡ Institute for Radiation Physics, Helmholtz-Zentrum Dresden - Rossendorf 01328, Dresden, Germany; § Departamento de Física Instituto de Geociências e Ciências Exatas, Universidade Estadual Paulista 13506-900, Rio Claro, SP, Brazil

**Keywords:** self-assembled monolayers, polyalanine α-helices, interfacial ordering, effective interaction model, chiral-induced spin selectivity

## Abstract

Self-assembled monolayers of polyalanine α-helices exhibit distinct structural phases with implications for chiral-induced spin selectivity. We combine scanning tunneling microscopy and theoretical modeling to reveal how chiral composition governs supramolecular organization. Enantiopure systems form hexagonal lattices, while racemic mixtures organize into rectangular phases with stripe-like features. Our interaction potentials derived from density-functional based tight binding calculations show that opposite-handed helix pairs exhibit stronger binding and closer packing, explaining the denser racemic structures. Crucially, we demonstrate that the observed STM contrast arises from antiparallel alignment of opposite-handed helices rather than physical height variations. These findings establish fundamental structure–property relationships for designing peptide-based spintronic materials.

## Graphical abstract

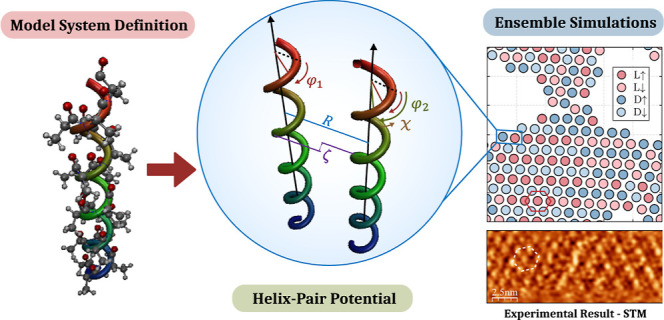

## 1 Introduction

Self-assembled monolayers (SAMs) play a fundamental role in modern materials science, with applications in nanotechnology, biosensing, and electronics.^1,2^ Among these, polypeptide SAMs are of significant interest due to their intrinsic ability to form chiral secondary structures, such as α-helices, which can introduce advanced functionalities because they can mediate spin-selective transport, a phenomenon known as the chiral-induced spin selectivity (CISS) effect.^3^ This effect enables efficient spin filtering without external magnetic fields or magnetic materials, thereby supporting advances in spintronics, where information is processed using electron spin rather than charge.^4–6^ Both experimental and theoretical work has demonstrated that helical polypeptides exhibit a pronounced CISS effect due to their rigid, chiral backbones, making them promising candidates for next-generation molecular spintronic devices.^7–12^

Despite these advances, fundamental questions regarding structure–property relationships in polypeptide SAMs remain open, particularly in the context of the CISS effect. A central issue is the mechanism of electronic transport through these systems, which lack the delocalized electronic structure common for organic conductors but yet exhibit highly efficient, spin-polarized charge transport.^13,14^ Furthermore, the driving forces behind the self-assembly process are not fully understood. Key questions include how the peptide sequence and specific functionalization influence the stability of different SAM phases and the kinetics of their formation, ultimately governing the supramolecular structure and its resulting electronic and spintronic properties.^15–17^

Among the wide variety of polypeptides, the polyalanine α-helix (αPA, see Figure 1) is particularly well-suited for studying self-assembly and the CISS effect, as it combines three key features: First, it adopts an α-helical structure, a common protein secondary structure stabilized by intrahelical hydrogen bonds.^18–20^ Second, its homopolypeptide nature, being composed solely of alanine residues, minimizes structural complexity and eliminates heterogeneous functionalization effects along the backbone. Last, alanine is the smallest functionalized amino acid, with only a methyl group as a side chain, which results in a minimal set of parameters necessary to specify the configuration of the helix. Moreover, this small side chain also ensures that self-assembled, parallel-aligned αPA molecules can pack densely due to reduced steric hindrance compared to polypeptides composed of larger amino acids.

**Figure 1:**
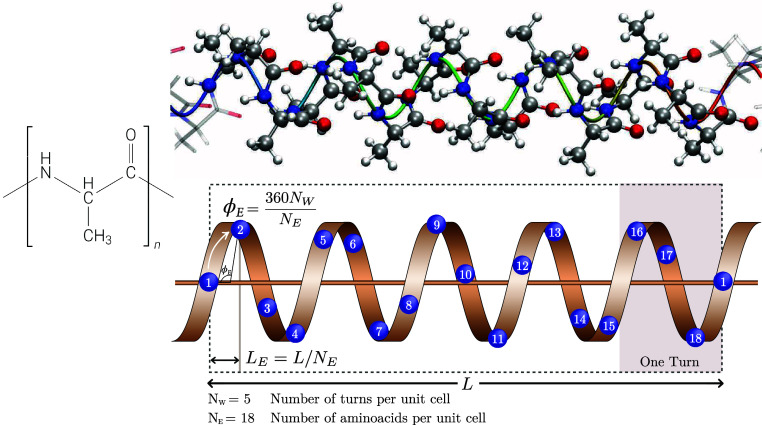
Chemical structure of polyalanine (left) and schematic representation of a polyalanine α-helix (right).

Recently, films of enantiopure right-handed αPA (L-PA) and racemic mixtures of right- and left-handed αPA (DL-PA) molecules formed on highly ordered pyrolytic graphite (HOPG) as well as on Al_2_O_3_/Pt/Au/Co/Au substrates were investigated under ambient conditions.^13,14,21^ In these works, both structural properties and spin-resolved electronic conduction properties were studied using scanning tunneling microscopy (STM) and scanning tunneling spectroscopy (STS), respectively. The latter revealed CISS magnetoresistance (CISS-MR) for all observed systems, confirming the spin-selectivity properties of αPA SAMs. In particular, it was found that the CISS-MR reached approximately 75% for chemisorbed hexagonal phases but dropped to around 50% for other phases. Additionally, the CISS-MR of chemisorbed molecules on Al_2_O_3_/Pt/Au/Co/Au substrates was up to 10% higher than that of physisorbed molecules on HOPG.^21^ Furthermore, the STS measurements confirmed that the α-helical conformation was preserved in both enantiopure and racemic films, with nearly identical HOMO–LUMO gaps (≈3.4 eV).^13^ The STM characterization of the different αPA SAMs revealed that for enantiopure L-PA on HOPG the adsorption resulted in a hexagonally close-packed (hcp) arrangement. In contrast, two distinct phases were observed for racemic DL-PA films. On the one hand, an hcp structure with identical characteristics to the enantiopure L-PA system was detected (Figure 2a, left). On the other hand, a novel phase with a rectangular unit cell has formed (Figure 2a, right).^14^ While STM height profiles revealed nearly uniform heights for the hcp structure (Figure 2, blue line) and along the shorter axis of the rectangular phase (Figure 2, red line), alternating apparent heights of about 3.0 Å along the longer axis were observed (Figure 2, black line). This apparent height difference reduces to about 2.5 Å four line scans diagonally to the rectangular cell (Figure 2, green line), suggesting that an αPA Helix is positioned in the center of the cell. Furthermore, it was found that these dimer phases exhibited a 25% higher packing density compared to the hcp structure, which comes along with a reduced distance of ≈0.4 nm between adjacent STM maximum features. This observation is consistent with Wallach’s rule, which states that racemates can form denser structures than enantiopure systems.^22^ Similar phases and lattice parameters were found also for chemically adsorbed αPA molecules on Al_2_O_3_/Pt/Au/Co/Au substrates,^21^ suggesting that the film properties are dominated by the intermolecular features rather than the substrate. For these systems, it is assumed that the helices are perpendicular to the substrate surface. In contrast, substantial tilt angles are known for chemisorbed peptide films on gold, where directional anchoring can enforce an inclined geometry, as observed for cysteine-terminated α-helices with reported tilt angles of about 40°.^8,23^

**Figure 2:**
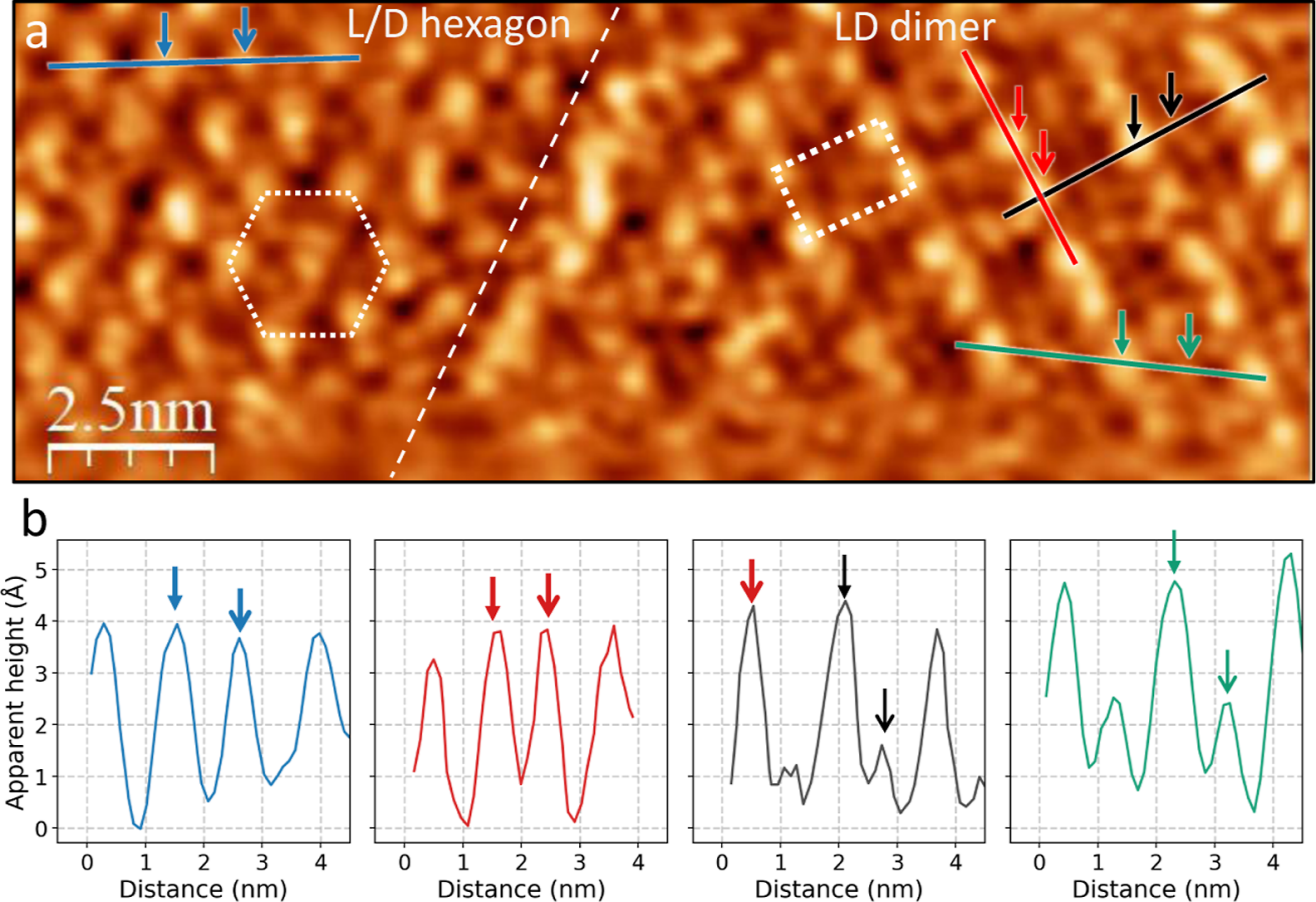
(a) STM image of a self-assembled film of racemic LD-αPA (LD-PA) on HOPG, showing the hexagonal phase of enantiopure L/D-PA (left) and the dimer phase of LD-PA (right). (b) Height profile taken along the colored lines in (a), showing the regular spacing and equal apparent heights in the hexagonal phase (blue arrows, left), variations in spacings but equal apparent heights along the parallel row (red arrows, middle-left), and regular spacing but differences in apparent height of adjacent rows within the dimer phase (green and black arrows, right).

The experimental observation of closer packing in the racemic mixture was interpreted assuming that intermolecular hydrogen bonds stabilize the individual phases, particularly the dimer phase.^21^ Although this reasoning provides a phenomenological understanding, a detailed atomistic picture of the intermolecular interactions that stabilize the various SAM phases is still lacking. For example, the specific nature of such intermolecular hydrogen bonds remains unclear. A deeper knowledge of such interactions, however, is crucial, since it might alter the electronic structure properties of αPA in SAMs compared to an isolated helix. In particular, the effect on the local charge distributions within the structure is important, since it has been recently shown experimentally that the orientation of the dipole can flip the sign of the CISS-MR.^24^ Hence, computational work addressing the structural properties of αPA or similar polypeptides at the molecular scale is of high value to better understand the individual interactions between neighboring αPA molecules in SAMs. Therefore, the here presented work aims to complement the experimental findings with theoretical insights into the intermolecular interactions within self-assembled structures of αPA. Our goal is to identify the specific interactions responsible for experimentally observed effects, such as the formation of hcp and rectangular phases and the molecular offsets within them. For clarity, we provide a concise flowchart in the Supporting Information that summarizes the sequential steps of our theoretical approach, from the isolated-helix model to the simulation of self-assembled films and their comparison to STM observations.

## 2 Methodology

### 2.1 Simulation of Interaction at Atomic Scale

To assess the geometrical properties of both isolated helices and helix pairs, a description of the interactions between all particles is required. For this purpose, we selected the self-consistent charge Density Functional based Tight Binding (SCC-DFTB) method, an approximate Kohn–Sham scheme.^25^ In contrast to classical force fields, SCC-DFTB is less empirical and directly provides the electronic structure of the system. While this electronic structure information is not the primary focus of the present work, it is crucial for future explorations of local dipole moments along the backbone, electronic transport properties, and the assessment of the CISS effect. The SCC-DFTB computations were performed using the DFTB+ software (version 20.1) with the mio-1–1 parameter set.^26^ Moreover, dispersion was included using the universal force field parameters of Rappé et al.^27^ While full geometry optimization was performed for isolated α-PA helices, single-point energy calculations were conducted for helix pairs. We note that more advanced DFTB variants and biomolecular parametrizations are also available and have been shown to improve the description of noncovalent interactions.^28^

In all computations, the helices were modeled as ideal, infinite structures using periodic boundary conditions along the helical axis. Neglecting the termination groups is a valid approximation in cases where helices are physisorbed so that intermolecular interaction dominates the formation of SAM structures, such as αPA on HOPG. Moreover, this approach suppresses termination effects such as the interaction of global dipole moments, and thus limits the interaction to local intermolecular interactions. Instead, considering infinite systems incorporates additional symmetry and, hence, reduces the degrees of freedom to a limited number of parameters, enabling a more sophisticated analysis of local interactions. It is important to note, however, that for helix pairs or ensembles the use of periodic boundary conditions restricts the configurations to parallel or antiparallel alignments.

### 2.2 Symmetry of Isolated α-PA Helices

In proteins and polypeptides, the α-helix is a well-known secondary structure, where the polypeptide backbone forms a helix stabilized by hydrogen bonds between the carbonyl group of one amino acid and the amide hydrogen of another one that is four residues away.^18,19^ This results in a structure in which each amino acid residue corresponds to a turn of ϕ_*E*_ = 100° and a translation of *L*_*E*_ ≈ 1.49 Å along the helical axis. Hence, 3.6 amino acids form a full turn with a pitch of approximately 5.4 Å.^20^ In an α-helix structure the functional groups of the individual amino acids face outward and therefore dominate the interaction with the environment.

To model this helical structure, we consider a unit cell of 18 alanine residues over five turns, with a total length along the helix direction of *L* ≈ 26.8 Å (Figure 1). That way, the unit cell exhibits redundancy, as a simultaneous rotation by ϕ_*E*_ and translation by *L*_*E*_ maps the molecular structure onto itself.

### 2.3 Symmetry Properties of Helix Pairs

To study the interaction between isolated helix pairs, a set of variables that uniquely defines all possible pair configurations must be chosen. To sample the interaction potential as densely as possible while conserving computational resources, it is important to avoid redundancy in the parameter space (i.e., not storing two configurations that represent the same atom arrangement due to symmetry). It is therefore meaningful to make use of the symmetry aspects to reduce the parameter space of the pair interaction.

While for a single isolated helix the absolute direction or handedness is unimportant, for helix pairs the relative alignment of the second helix with respect to the first must be considered. In addition to the helices’ handedness (right- or left-handed), the relative orientation of molecular features, such as the direction of the carbonyl groups, impacts the interhelical interaction. Consequently, four distinct alignments must be considered, which are summarized in Table 1: (1) equally handed, parallel alignment (EP), (2) equally handed, antiparallel alignment (EA), (3) oppositely handed, parallel alignment (OP), and (4) oppositely handed, antiparallel alignment (OA).

**Table 1:**
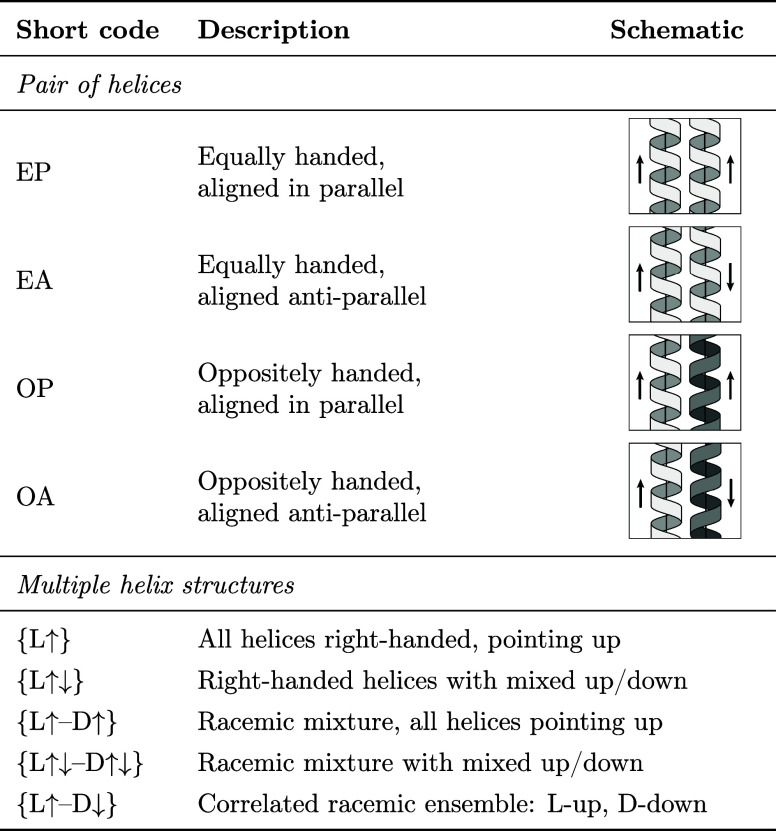
Nomenclature for Helix Pairs and Multi-Helix Ensembles (SAM)

Each of these four interaction types is specified by four continuous variables: the interhelical distance *R*, the rotation angles of the helices φ_1_ and φ_2_, and the relative lateral offset ζ. While the distance *R* is intuitively given as the separation between the two helix axes, the choice of how to define the angular variables φ_1_, φ_2_ and the shift parameter ζ, requires a more careful consideration. Without loss of generality, the first helix is considered to be right-handed with the carbonyl group pointing in the positive *z* direction, which is referred to as up-orientation. This choice defines the relative coordinate system, which specifies the signs of φ_1_, φ_2_ and ζ. Their specific value is defined based on the positions of one of the 18 symmetry-equivalent nitrogen atoms within the repeat units of each of the helices. For the first helix, we select the nitrogen atom closest to the interhelical distance vector. This choice confines φ_1_ to a narrow range around the axis of minimal separation, specifically (−10°, +10°). For the second helix, the reference nitrogen atom is chosen such that its axial displacement relative to the selected nitrogen of the first helix is positive but does not exceed *L*_*E*_. This condition allows φ_2_ to vary freely over 0° to 360°, while restricting the lateral shift ζ to the interval 0 ≤ ζ < *L*_*E*_, ensuring only unique configurations along the helical axis are considered.

Instead of considering the absolute rotation angle φ_2_ to specify a pair configuration, we introduce a more convenient parameter

$$\chi =\varphi_{1}-\mathrm{h̃}\varphi_{2}$$

where *h̃* = +1 if both helices have equal handedness, and *h̃* = -1 if their handedness is opposite. For helices of equal handedness, χ is the angular difference between their reference atoms; for opposite handedness, it is the angular sum. This definition arises from the inherent screw symmetry of the helices: if two helices of equal handedness are simultaneously rotated by integer multiples of ϕ_*E*_ = 100°, the resulting configuration is equivalent to the original one, differing only by the choice of the reference atom, as now another nitrogen atom of the first helix is closest to the interhelical distance vector. In the case of helices with opposite handedness, the same invariance holds if the helices are rotated by equal amounts in opposite directions.

Formally, the parameter χ may be chosen within any interval covering a full rotation of 360°, such as (0°, 360°) or (−180°, 180°). In our case, however, we restrict the range differently, making use of an additional symmetry consideration: For two helices that are aligned in parallel (not antiparallel), the pair interaction obeys not only the helical symmetry discussed above but also an additional invariance that arises from the fact that the two helices are identical and interchangeable. In fact, the choice of which helix is labeled first and which second is arbitrary. Hence, there exists a transformation between the two choices that formally changes the first and the second helix. For parallel aligned helices (equal or opposite handedness), this transformation corresponds to a reflection through the point (0°, –ϕ_*E*_/2) in the (φ_1_, χ) plane. To explicitly include this symmetry in our analysis, we select the range of χ such that the inflection point lies in the middle of the chosen interval. This is achieved by restricting χ to the interval [−230°, 130°], which spans a full rotation of 360° but is centered on the symmetry point at $\frac{1}{2}\phi_{E}=-50^{{}^{\circ}}$ . In this way, equivalent configurations related to labeling the helices in reverse order appear symmetrically within the parameter space, which simplifies both visualization and interpretation of the results.

### 2.4 Ensemble Simulation

To study low-energy structures based on pair interactions between all involved helices, we considered ensembles of 160 helices in a square simulation box with periodic boundary conditions. Each micro state of the ensemble is then defined by the 2D position of the helix centers (*x*_*i*_ and *y*_*i*_), the rotation angles (φ_*i*_), and the vertical shifts (*z*_*i*_) of all helices. All of these parameters are defined with respect to a global coordinate system. Again, the rotation angle and height displacement are specified with respect to one of the 18 symmetry-equivalent nitrogen atoms. In addition, each helix has a handedness *h*_*i*_ (1 for right-handed and −1 for left-handed) and a direction *d*_*i*_ (+1 for up, −1 for down). Based on these parameters, the contribution from each pair interaction can be obtained by transforming into the proper reference system through translation, rotation and mirroring such that one helix is right-handed point up.

To assess the phases that can be formed from parallel and antiparallel aligned L-PA and/or D-PA, we considered enantiopure ensembles as well as racemic mixtures, with purely up-aligned systems and mixed up-and-down alignments. This results in four fundamental ensembles to which we refer as {L↑}, {L↑↓}, {L↑-D↑}, and {L↑↓-D↑↓}. In addition, we consider a specific case where all right-handed helices are aligned upward while left-handed ones are aligned downward, which we refer to as {L↑-D↓}. Table 1 summarizes the individual ensembles.

Low-energy structures of the considered ensembles were obtained from random initial configurations using simulated annealing with the Metropolis algorithm. To explore the configuration space, we applied four ergodic transformations: in-plane displacement, vertical displacement, rotation, and swaps of helices with distinct chiralities or orientations. These moves were randomly selected in a 3:3:3:1 ratio to ensure efficient sampling. The simulations used a pseudoexponential annealing schedule: starting at *T*_start_ = 10 000 K, we performed 1.5 × 10^6^ Metropolis steps at each temperature, followed by a temperature reduction by a factor of τ = 0.9 until the simulation temperature reached a value below *T*_end_ = 0.01 K.

In this study, we focus only on the properties of the low-energy configurations obtained at the end of the simulation. The specific paths to these states are not physically meaningful, because relative diffusion rates are not reflected in the chosen ration between update types. However, some insights can be gained from the simulation process, particularly from the specific heat, see Figure S1, whose features can be attributed to the formation of structural properties observable in the final films. For completeness, representative results from the simulated annealing simulations are provided in the Supporting Information, Section S1.

## 3 Results and Discussion

### 3.1 Pair-Interaction Potentials

#### 3.1.1 Distance-Dependent Interaction Energy at Frozen Relative Orientation

As defined in Section 2, the interaction energy between two helices depends on four continuous geometric parameters: the interaxial distance *R*, the azimuthal angle φ_1_ of the first helix, the relative angle χ (defined as the sum or difference of the two azimuthal angles, depending on handedness), and the relative vertical offset ζ. Additionally, different combinations of handedness and axial direction result in the four distinct interactions: EP, EA, OP, and OA. The four-dimensional parameter space (*R*, φ_1_, χ, ζ) makes direct visualization of the interaction energy challenging. To reduce complexity, we begin by examining the dependence on *R* while freezing the other degrees of freedom, thereby isolating distance regimes where relative orientation significantly influences the interaction.

At large separations, local contacts between functional groups—whose spatial arrangement is determined by relative orientation—play a minor role, and the interaction energy approaches zero. In contrast, at short to intermediate distances, steric complementarity and the possibility of interdigitation dominate the interaction. To explore these effects, we computed distance-dependent binding energy profiles for a comprehensive set of fixed relative orientations. Specifically, for each combination of φ_1_, χ, and ζ (10 × 18 × 10 = 1800 configurations), we evaluated *E*_bind_(*R*) for *R* ranging from sterically forbidden overlaps up to 20 Å, beyond which interactions are negligible. The binding energy *E*_bind_(*R*) is obtained as the difference between the total energy of the dimer and twice the total energy of an isolated helix.

Figure 3a shows binding energy *E*_bind_(*R*) curves for four selected relative orientations in EP alignment (colored lines), with the corresponding (φ_1_, χ, ζ) values indicated in the legend. These selections illustrate the diversity in both optimal binding distance *R*_opt_ and minimum energy *E*_min_ arising from differences in relative orientation. The *E*_bind_(*R*) profiles are overlaid on the complete set of equilibrium distances *R*_opt_ and minimum energies *E*_min_ for all 1800 relative orientations (gray dots). Three key observations emerge: (i) For all configurations, the energy curves exhibit a well-defined minimum, with equilibrium spacing between 8.6 Å and 10.3 Å; (ii) The depth of the corresponding energy minima varies from −1.07 eV to −0.25 eV; (iii) At larger distances (*R* > 10.5 Å), the curves converge and orientation-dependent differences become negligible, confirming that angular effects dominate primarily in the near-contact regime.

**Figure 3:**
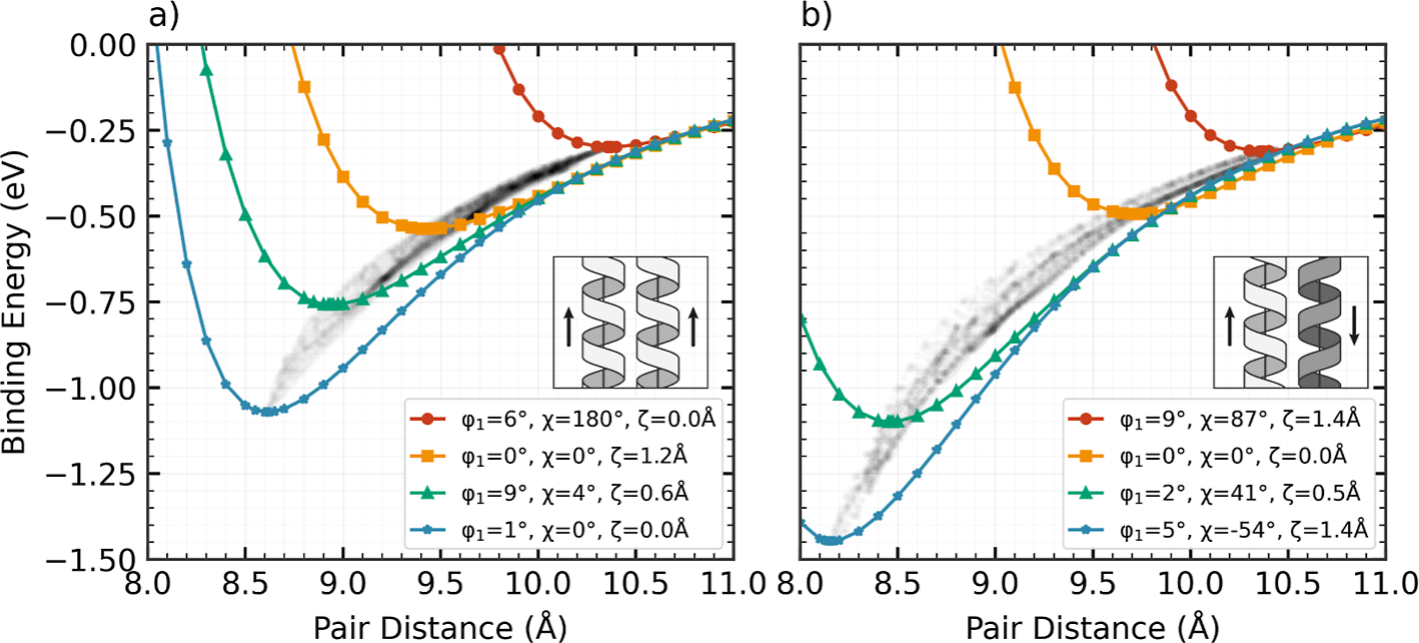
Distance-dependent binding energy profiles for representative configurations under fixed orientation parameters (φ_1_, χ, ζ). (a) Four cases from the OA class; (b) Four cases from the EP class. Gray curves in the background correspond to all other sampled configurations, illustrating the overall variability across the orientation space. The legends indicate (φ_1_, χ, ζ) values for each highlighted curve. These results demonstrate that opposite-handedness (OA) enables stronger binding and closer contact than same-handedness (EP) arrangements.

Figure 3b presents corresponding results for OA alignment, where helices possess opposite handedness and are oriented antiparallel. While the overall shape of the energy curves remains similar to EP alignment, two notable differences emerge. First, the minimal equilibrium spacing shifts toward smaller values, with *R*_opt_ reaching as low as 8.16 Å. Second, the interaction strength is significantly enhanced: the most favorable configurations exhibit binding energies up to −1.45 eV, approximately 400 meV deeper than the strongest EP configurations. These findings indicate that opposite-handed arrangements enable closer approach and stronger stabilization, consistent with Wallach’s rule for isolated αPA strands.

The remaining interaction possibilities, EA and OP, are presented in the Supporting Information, where similar qualitative trends are observed (Figure S3). For EA alignment, minimal distances for frozen orientations range from 8.5 Å to 10.3 Å, with corresponding binding energies between −1.04 eV and −0.25 eV, whereas OP configurations exhibit slightly smaller equilibrium distances up to 8.3 Å, accompanied by stronger binding energies reaching up to −1.30 eV. These results further support that relative handedness and orientation govern both equilibrium spacing and interaction strength between helices. For better comparability of the energy ranges and obtained equilibrium distances under frozen azimuthal angles and offsets, Figure S4 overlays the boundary of the obtained distributions.

For interaxial distances larger than 11 Å, the interaction energy approaches zero, independent of the specific frozen configuration within a given alignment, and is nearly identical across all four classes (EP, OA, EA, and OP). Based on this observation, the long-range portion of the computed energy curves was fitted to a 1/*R* ^6^ term and subsequently extrapolated to account for distances beyond the sampled range.

#### 3.1.2 Dependence on Relative Offset

We now analyze the interaction as a function of the relative axial displacement between two helices. For each combination of φ_1_ and χ, the relative vertical offset ζ was varied from 0 to *L*, while the interaxial distance *R* was always chosen to correspond to the minimal value for the considered set of φ_1_, χ, and ζ. This approach allows visualization of the effect of steric repulsion of the functional methyl groups, which can either permit or prevent interdigitation.

Results for EP and OA configurations with φ_1_ = 0° and χ = 0° are shown in Figure 4 as circles, where the horizontal axis corresponds to the optimal pair distance and the vertical axis to offset ζ. The color code indicates the binding energy for each configuration. Additionally, the graphs feature the distance and energy for each offset ζ where φ_1_ and χ yield minimal binding energy (triangles). Due to the helical symmetry of each helix, a shift of *L*_*E*_ is equivalent to a rotation of 100°. Therefore, the curves presented in Figure 4 represent configurations with φ_1_ being multiples of 20◦ but displaced by the corresponding lateral shift.

**Figure 4:**
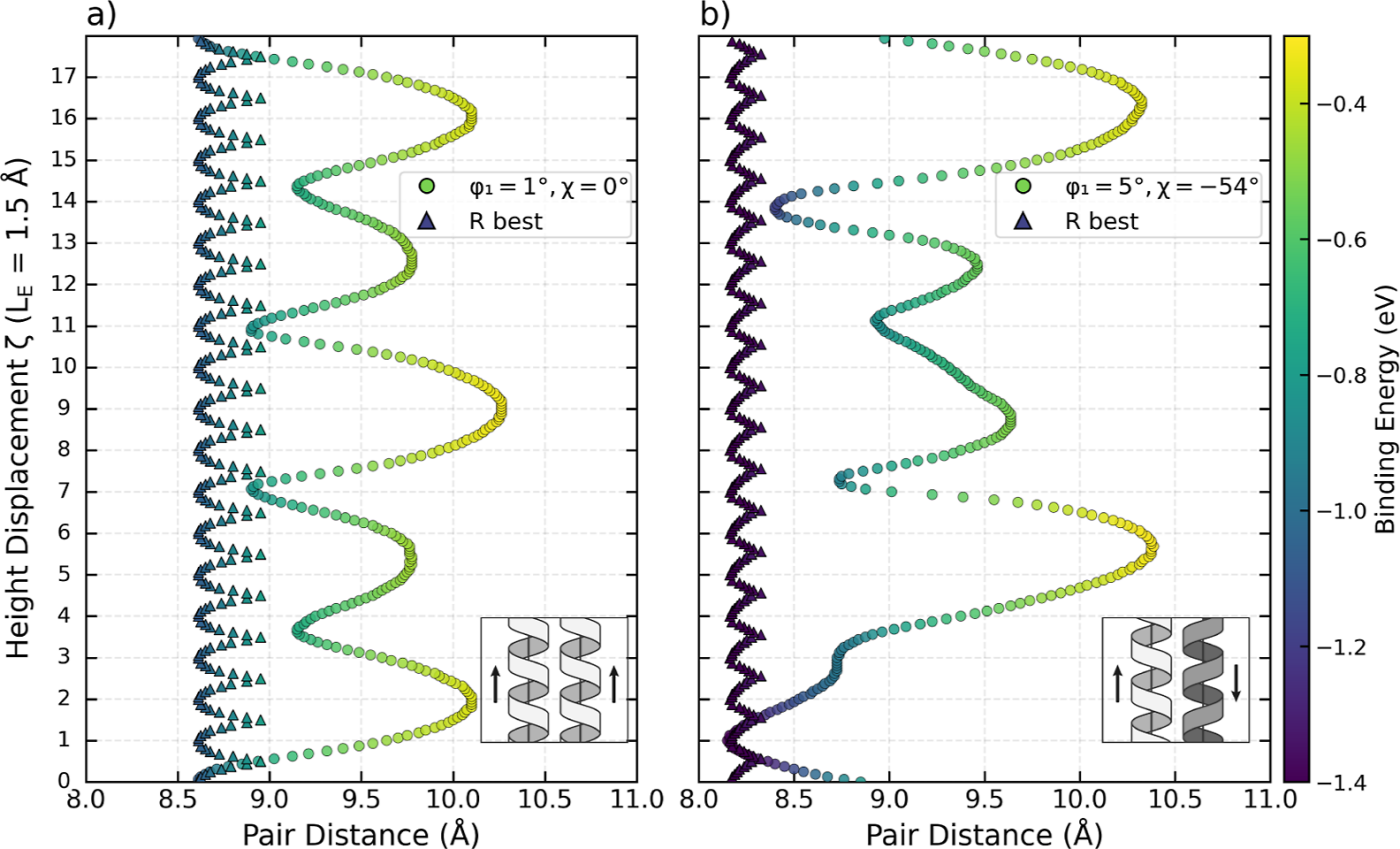
Optimized pair distance *R* for fixed φ_1_ and χ as a function of the relative vertical offset ζ for EP (a) and OA (b) alignments. Each colored circle represents the binding energy for a given φ_1_-χ combination, with the color scale indicating the magnitude of the interaction energy (blue: stronger binding, red: weaker binding). Triangles indicate the minimal pair distance *R*_best_ for each ζ, corresponding to the most favorable angles φ_1_ and χ. The oscillatory dependence on ζ highlights the critical role of axial registry in achieving optimal interdigitation and binding strength.

For EP alignment (Figure 4a), the closest configuration is obtained for zero relative offset and vanishing angles φ_1_ and χ. This result can be explained by the fact that for equally oriented, parallel helices of the same handedness, the helical structure adopts an interdigitated configuration. Upon a lateral shift of half the pitch, interdigitation is lost due to steric repulsion between helix backbones. This repulsion is strongest when methyl groups face each other, occurring in configurations such as (φ_1_ = 0°, χ = 180°, ζ = 0.0 Å) or equivalently (φ_1_ = 0°, χ = 0°, ζ = 9 *L*_*E*_) due to symmetry. As shown in Figure 4a, this configuration corresponds to the largest *R*_opt_. Moreover, due to additional symmetry for parallel-aligned helices, the graphs exhibit mirror symmetries with respect to interdigitate (ζ = 0) and noninterdigitate configurations (ζ = 9 *L*_*E*_).

For the OA system (Figure 4b), similar behavior is observed. Upon shifting the two helices for fixed angles, the optimal distance and corresponding binding energy oscillate between interdigitated configurations with smaller optimal distances and lower binding energies, and noninterdigitated configurations with larger distances up to 10.5 Å. In contrast to the EP case, the curve lacks symmetry due to the missing symmetry of antiparallel aligned helices. Furthermore, configurations of the smallest and largest *R*_opt_ are not related via a 180° rotation of one helix, as different helix orientations result in different functional group arrangements.

Similar observations for EA and OP cases, including symmetry properties for parallel alignment and closer distances for opposite-handed pairs, are presented and discussed in the Supporting Information (Figure S5). In particular, the additional symmetry with respect to a shift of 0 Å and 9 *L*_*E*_ for parallel alignment, which is missing for antiparallel alignment is evident.

#### 3.1.3 Angle Dependence

Having analyzed the interaction potential of helix pairs with respect to distance and offset at fixed angles, we now consider the energy landscape regarding angular orientation. As presented in Section 2, symmetry properties of the configuration space allow φ_1_ to be limited to −10° to 10° while χ spans a full rotation of 360°. To incorporate the additional symmetry point for parallel-aligned helices at (−Φ_*E*_/2), we choose the relative angle parameter χ (angle difference for same-handedness helices and angle sum for opposite-handedness helices) in the range −230° to 130°.

Figure 5 shows the obtained binding energies with corresponding values of *R*_opt_ and ζ_opt_ in heat map format. For the EP configuration (Figure 5a), the heatmaps reveal how interaction energy, equilibrium distance, and relative offset depend jointly on angular variables φ_1_ and χ. The binding energy map (top) exhibits pronounced two-dimensional features, indicating both angular parameters substantially shape the interaction. Smooth variation across both φ_1_ and χ with binding energy variations up to 0.6 eV demonstrates that in EP alignment, orientation cannot be reduced to a single dominant parameter but emerges from genuinely two-dimensional angular dependence.

**Figure 5:**
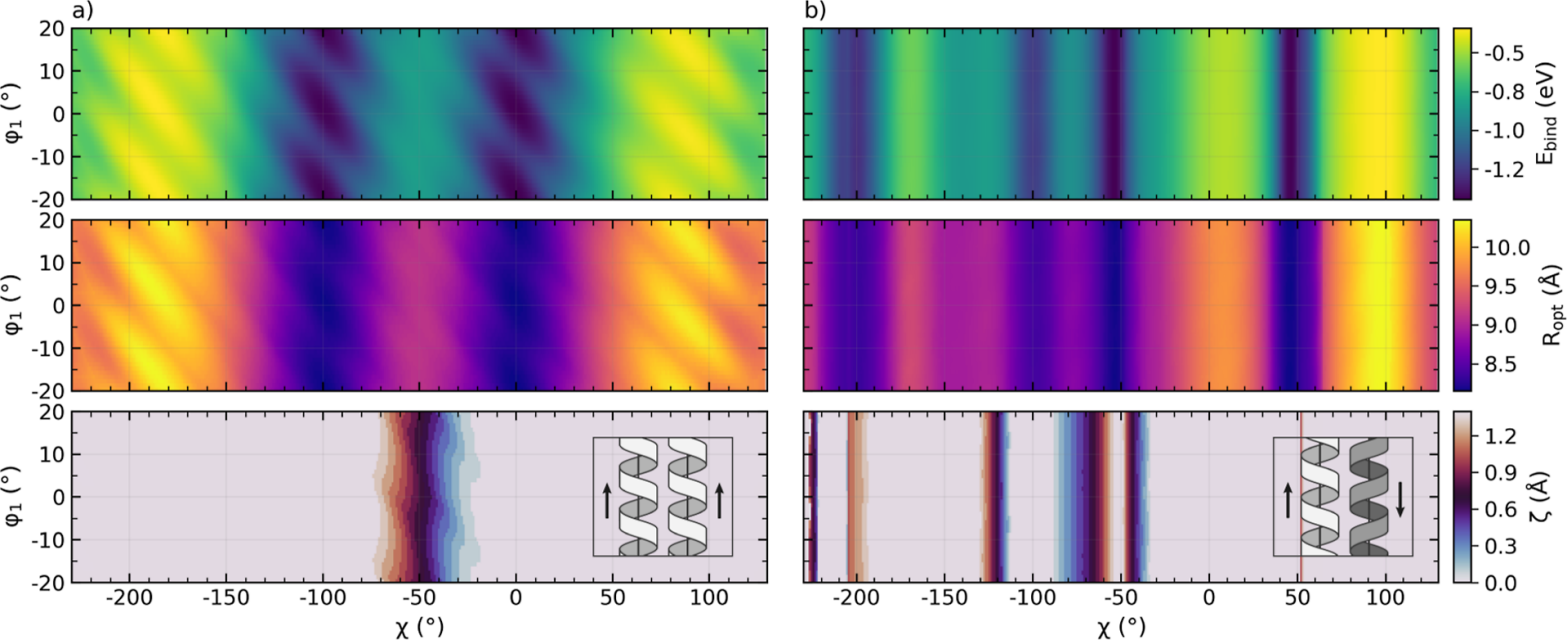
Heatmaps of binding energy (top), equilibrium distance *R*_opt_ (middle), and relative offset ζ_opt_ (bottom) as a function of angular parameters φ_1_ and χ for EP (a) and OA (b) configurations. The EP configuration shows complex 2D dependence on both angles, while the OA configuration depends primarily on the relative sum χ, revealing a fundamental difference in how handedness dictates the interaction landscape.

Symmetry-related structures can be identified at inflection points around φ_1_ ≈ −10° and 0° combined with χ = −50°, 130°, and −230°. These equivalent points arise from intrinsic helical symmetries and involve compensating shifts in axial offset by *L*_*E*_, as reflected in the offset map (bottom). A distinct global minimum is observed near φ_1_ ≈ 1°, χ ≈ 1° (and correspondingly near φ_1_ ≈ −1°, χ ≈ –99°), with optimal distance *R*_opt_ ≈ 8.7 Å and zero offset (ζ_opt_ = 0).

In contrast, the OA configuration (Figure 5b) shows markedly different features. Here, binding energy maps are dominated by stripe-like features aligned along φ_1_, demonstrating interaction insensitivity to the absolute value of φ_1_. Instead, the dominant dependence is on the relative sum χ = φ_1_ + φ_2_, consistent with opposite helicity. Due to antiparallel alignment, inversion symmetry with respect to the configuration (φ = 0°, χ = −ϕ_*E*_ = −50°) present in the EP case is lost. Instead, two preferential orientations with binding energies of about −1.44 eV stand out at χ ≈ 46° and χ ≈ –54°, with additional local minima around χ ≈ −100° and −200°. Equilibrium distances and offsets reflect these patterns, with sharp transitions in ζ_opt_ aligning with changes in favorable angular configurations.

Corresponding analyses of EA and OP interactions are shown and discussed in the Supporting Information, where qualitatively similar observations are found: well-defined global minimum configurations for EA alignment Figure S6a and stripe-like features aligned along φ_1_ due to the opposite-handedness and inversion symmetry in the (φ_1_, χ)-plane with respect to (0°, −50°) because of the parallel alignment for the OP system (Figure S6b).

In summary, the pair interaction analysis reveals that OA and OP configurations, involving helices of opposite handedness, consistently allow closer approach (smaller *R*_opt_) and stronger binding (more negative *E*_min_) than EP or EA configurations. This fundamental difference in pairwise stability, dictated by relative handedness and orientation, is the key factor governing structural properties of larger self-assembled films discussed in the following section.

### 3.2 Global Minimum Configurations of Helix Pairs

After analyzing the dependence of the interaction potential on distance, offset, and angular orientation, we now aim at identifying which specific interactions stabilize the most favorable helix arrangements.

Figure 6 displays selected configurations of EP (a) and OA (b) helix pairs, which correspond to local minima of the interaction potentials presented in Section 3.1. The representations show the complete unit cell comprising 18 alanine residues over five helical turns of each helix. For better visualization of the individual handedness and up/down orientation, the helical backbone is indicated by a coil. Furthermore, the intrahelical hydrogen bonds stabilizing the α-helical structure are shown as orange dashed lines. To characterize the intramolecular interaction, the functional methyl groups with distances to groups of the other helix of less than 4.0 Å are highlighted by transparent yellow spheres. The corresponding plots for EA and OP are presented in Supporting Information (Figure S7).

**Figure 6:**
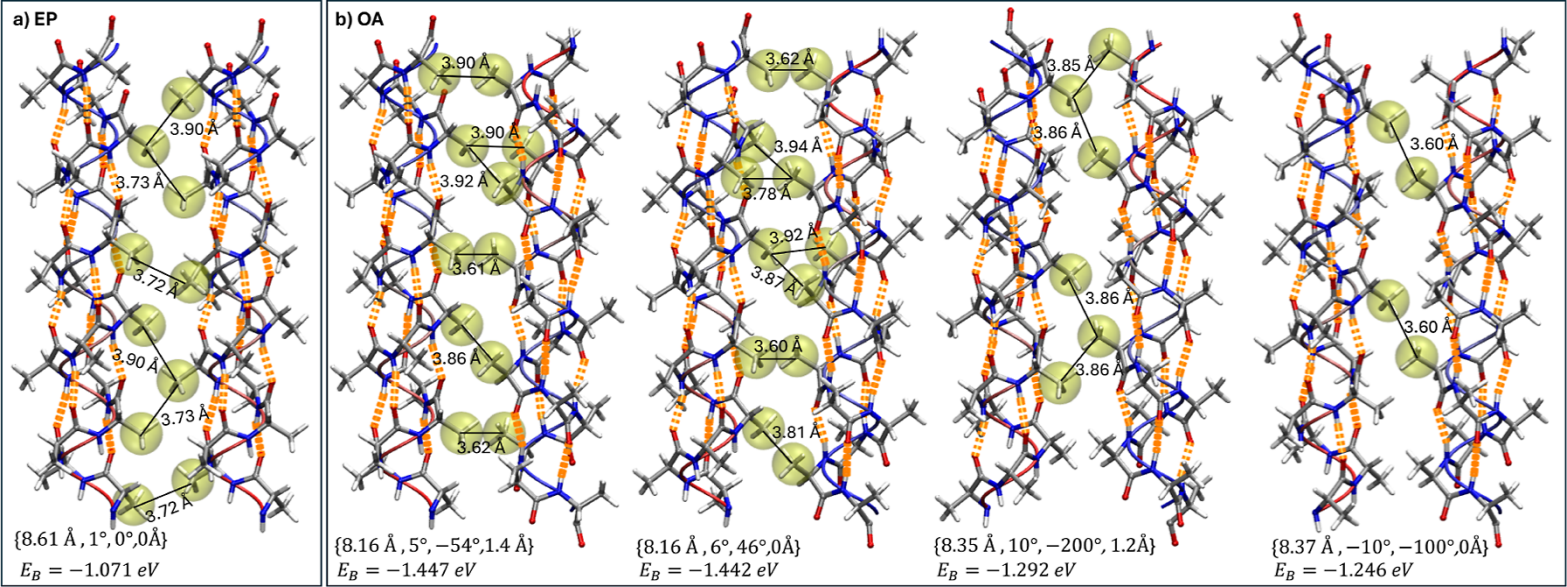
Local Minimum configurations of EP (a) and OA (b) helix pairs, where for better recognition the helical backbone is represented as a coil. The specific relative configuration parameters *R*, φ_1_, χ, and ζ as well as the corresponding binding energies *E*_*B*_ are given below each configuration. Intrahelical hydrogen bonds stabilizing the α helix are indicated in orange. Methyl groups with interhelical distances smaller than 4.0 Å are highlighted by yellow spheres with the individual distances given in the figure.

For the EP system, the global minimum corresponds to a configuration with φ_1_ = 1°, χ = 0°, and ζ = 0.0 Å which can be interpreted as if one helix is only horizontally displaced from the other. This consideration yields an explanation why it constitutes the energetically most favorable configuration as it allows the best way to interdigitate the helices, allowing for dense packing. Any relative rotation or offset of one of the helices would lead to unfavorable interactions between functional groups, increasing the helix–helix distance and thus reducing the overall binding strength. As shown in Figure 6a, in this confirmation there are six methyl–methyl contacts with distances ranging from 3.7 Å to 3.9 Å. In line with previous quantum-chemical analyses of methyl–methyl contacts published by Dutta et al.,^29^ these intermolecular distances are consistent with the presence of weak but attractive dispersion–driven interactions with energies of approximately −0.02 to −0.07 eV per contact (−2 to −7 kJ mol^–1^).

For the global minimum of the OA interaction (Figure 6b, left), there are also six methyl–methyl contacts with distances smaller than 4.0 Å. Here, however, these distances are smaller than for EP and approach the ideal distance reported by Dutta et al.^29^ For the second most stable local minimum configuration (Figure 6b, middle-left), similar interactions are observed, resulting in a binding energy nearly identical to the global minimum. The remaining two local minimum configurations of the OA alignment (Figure 6b, middle-right and right), on the other hand, exhibit only four and two interaction points and thus a lower number of interhelical methyl–methyl distances smaller than 4.0 Å.

Comparing the structural properties of these minimum configurations and their related individual energies, the following conclusions are drawn: On the one hand, the local interaction of the closest motifs are between the nonpolar methyl groups. The distances of polar groups of different helices, such as the amide and the carbonyl group, are too large to form hydrogen bridges. Hence, the stabilizing interaction is of the van der Waals type rather than hydrogen bonding as previously assumed.^14^ As van der Waals interactions do not alter the electronic density of each helix, it can be concluded that the attractive interactions between helices do not disturb the electronic structure of each individual helix. This finding provides a justification that for studies focusing on electronic transport, the consideration of isolated single helices is sufficient. In particular, local dipole moments concluded from models of isolated helices are valid to describe the properties of helix bundles or films. On the other hand, the total binding energy is −1.0 eV up to −1.4 eV, i.e., up to 20 times larger than the optimal interaction between two nonbonded methyl groups. As only six or less of such interactions are present, it is concluded that the stabilization of the individual configuration is not due to a few specific local interactions but rather reflects the cooperative contribution of the full molecular geometry. This also includes dispersive interactions involving the atoms of the helical backbone itself, not only the side-chain methyl groups. This conclusion is strengthened by the fact that local minima of OA with only two close methyl group pairs (Figure 6b, right) shows stronger binding than the EP configuration with six of such interactions. In conclusion, the interaction between two peptide helices cannot be reduced to a single local motif, as any change in configuration simultaneously alters multiple attractive and repulsive contributions, reflecting the complexity and the frustrated nature of the interaction.

### 3.3 Low-Energy Configurations of Self-Assembled Films

After analyzing the interaction properties of isolated helix pairs, we now examine ensembles to investigate how these pairwise interactions manifest in larger assemblies. This allows us to assess whether optimal pair configurations are preserved in collective structures or whether frustration effects and packing constraints drive the system into alternative arrangements.

To investigate properties of low-energy arrangements that reflect features of experimentally observed self-assembled structures, we analyze structural aspects of configurations obtained from heuristic optimization using simulated annealing. Figure 7 shows representative 2D structures for each of the five ensembles considered, with notable features that will be discussed in detail.

**Figure 7:**
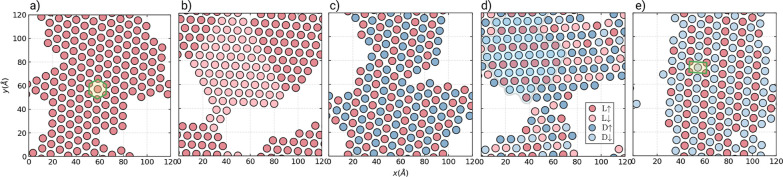
Representative configurations of self-assembled films for the different systems are defined in Table 1: (a) {L↑}, (b) {L↑↓}, (c) {L↑-D↑}, (d) {L↑↓-D↑↓}, and (e) {L↑-D↓}.

To complement the structural characterization, we performed statistical analysis over the best film configurations obtained from 100 independent runs for each system. We focus on structural properties, particularly the radial distribution function (RDF) and the relative orientation of nearest neighbors (RONN), by averaging over all 100 configurations. Considering that each ensemble consists of 160 helices in close packing with up to 6 nearest neighbors each, the statistical analysis includes over 30,000 individual pair arrangements, providing a solid basis for characterizing dominant film features.

#### 3.3.1 Structural Properties of Parallel Enantiopure Films

For the {L↑} system (all helices of equal handedness and parallel alignment), all pair interactions are of type EP. As shown in Figure 7a, simulations result in a hexagonal pattern. Analysis of the RDF shown in Figure 8a confirms perfect hexagonal alignment. Well-defined peaks occur at 8.6 Å, 14.9 Å, 17.2 Å, and additional positions. These ratios reflect characteristics of an ideal hexagonal lattice. Based on the nearest-neighbor distance *a*_0_ = 8.6 Å, the expected second- and third-nearest neighbor distances are precisely at $\sqrt{3}a_{0}=14.9Å$ and 2*a*_0_ = 17.2 Å, respectively. Comparing this finding with the pair interaction potential reveals that this distance of approximately 8.6 Å represents the lower boundary of the optimized distance between two isolated helices in EP configuration (see Section 3.1), indicating very dense packing.

**Figure 8:**
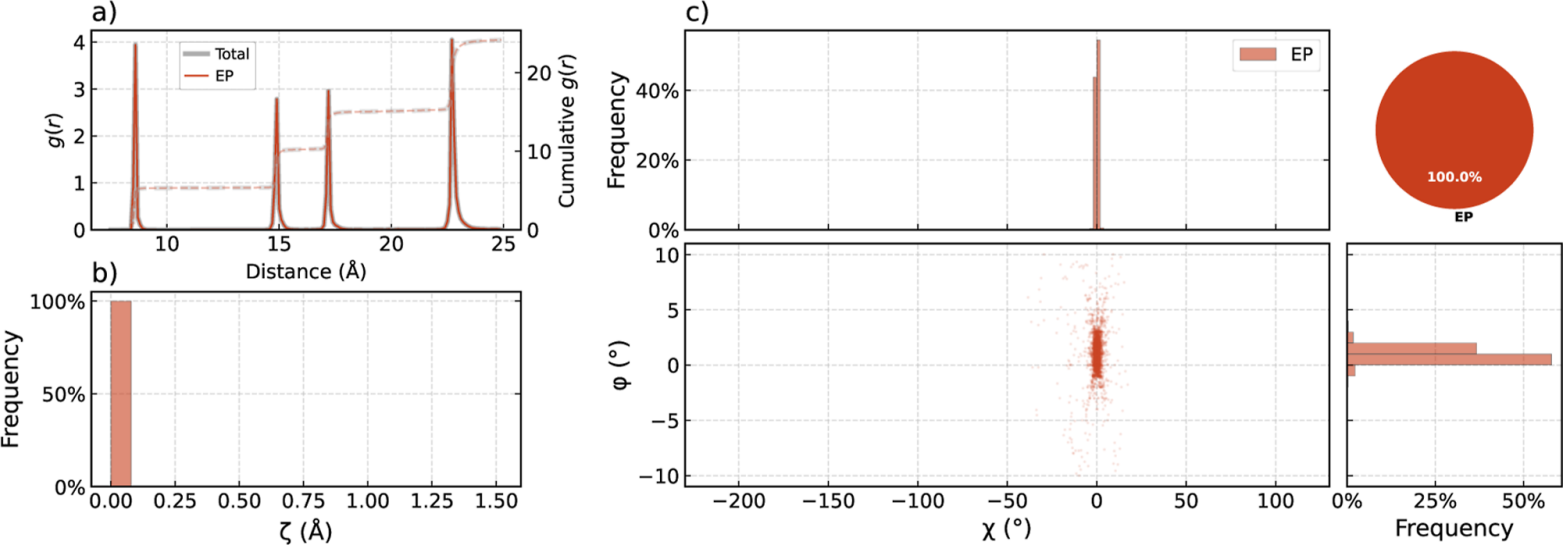
Structural analysis of the {L↑} system. (a) Radial distribution function (RDF) showing perfect hexagonal packing. (b) Distribution of the angle φ_1_ for nearest neighbors. (c) Joint distribution of the angle difference χ and relative axial offset ζ for nearest neighbors. The data confirms a frustration-free, homogeneous structure where every pair interaction is in its optimal EP configuration.

To characterize RONN, φ_1_, χ, and ζ of all pairs with distances smaller than 10 Å were statistically examined. Figure 8b shows the distribution of offset ζ, which is sharply peaked at 0 Å. Figure 8c presents the joint distribution of angle φ_1_ and relative angle χ as a scatter plot with projected histograms along the corresponding axes. The results demonstrate that essentially all nearest-neighbor contacts occur at χ ≈ 0° and ζ ≈ 0 Å, indicating a strict angular arrangement. This observation underlines the high degree of structural homogeneity in this ensemble and shows that dense hexagonal packing is realized exclusively through energetically optimal EP contacts. This frustration-free arrangement is only possible due to the fact that the helical symmetry of αPA reduces the value of φ_1_ to a range with repetition every 20°. Since in a perfect hexagonal lattice the angle between lattice vectors equals 60° (a multiple of 20°), a helix can adopt the optimal configuration with all its neighbors simultaneously.

To investigate the influence of up/down alignment on self-assembled structures, simulations of same-handed helices either oriented up or down were conducted ({L↑↓} system). From Figure 7b, one observes: (i) a densely packed overall hexagonal arrangement; (ii) demixing of differently oriented helices into individual domains; and (iii) straight domain boundaries. Observation (ii) can be explained as follows: Since a pure EP system forms frustration-less films, where each nearest-neighbor interaction is in its global minimum and any EA orientation is energetically less favorable than EP alignment with ζ = 0 and χ = 0 (see Section 3.1), the bonding energy of a helix is lowest when surrounded by six helices of the same alignment. For mixtures of up and down orientated helices, this causes the demixing. Observation (iii) can be understood by considering that at domain boundaries, each helix is in EA interaction with some of its neighbors. For straight boundaries, each helix is surrounded by four helices of the same direction (EP interaction) and two of opposite alignment (EA interaction). Nonstraight boundaries would require “corner points” with three neighbors of each type, which is energetically less favorable, leading to kink-free domain boundaries. Regarding observation (i), the cross-domain hexagonal structure can be explained by the fact that the most stable EA orientation occurs at 8.65 Å, similar to the EP lattice constant (see Section 3.1). That these EA arrangements are also in the best possible pair-interaction configuration is confirmed by RONN statistics in Figure S8, showing a narrow distribution at χ = −77° and ζ = 0.3 Å corresponding to the global-minimum arrangement of EA pairs. Due to boundary minimization, less than 6% of all nearest neighbor interactions are of EA nature.

We conclude that enantiopure films tend toward structures of parallel alignment with identical angular orientation and vanishing vertical displacement, resulting in hcp structures. This confirms experimental observations reported for enantiopure L-PA films.^14^ It is particularly worth noting that, in contrast to our theoretical system of infinite chains, experimental αPA is terminated by amine or carboxyl groups. Due to the chemical differences of these capping groups, constant-current STM measurements would result in apparent height differences. We therefore conclude that STM measurements also indicate parallel aliments in the enantiopure domains.

#### 3.3.2 Structural Properties of Films Formed from Racemic Mixtures

For the {L↑-D↑} system (racemic mixture, all helices up-aligned), Figure 7c indicates an overall hexagonal structure where right- and left-handed helices are arranged in a line pattern. Analysis of the RDF (Figure 9a) reveals that, in contrast to the enantiopure systems, the lattice is not perfectly hexagonal. Instead, a difference in nearest neighbor distances between helices of the equal and opposite handedness is observed, indicated by two-peak features in the RDF. Deconvolution of the RDF into individual EP and OP interactions shows a 2:1 ratio, which can be explained by the fact that in the line-shaped configuration each helix has two neighbors of equal handedness and four neighbors of opposite handedness. Because OP interactions allow for denser packing (see Section 3.1), the hexagonal lattice compresses perpendicular to the lines, enabling closer distances for OP interactions while maintaining the EP distance. The peak shapes of the RDF at larger distances confirm this interpretation.

**Figure 9:**
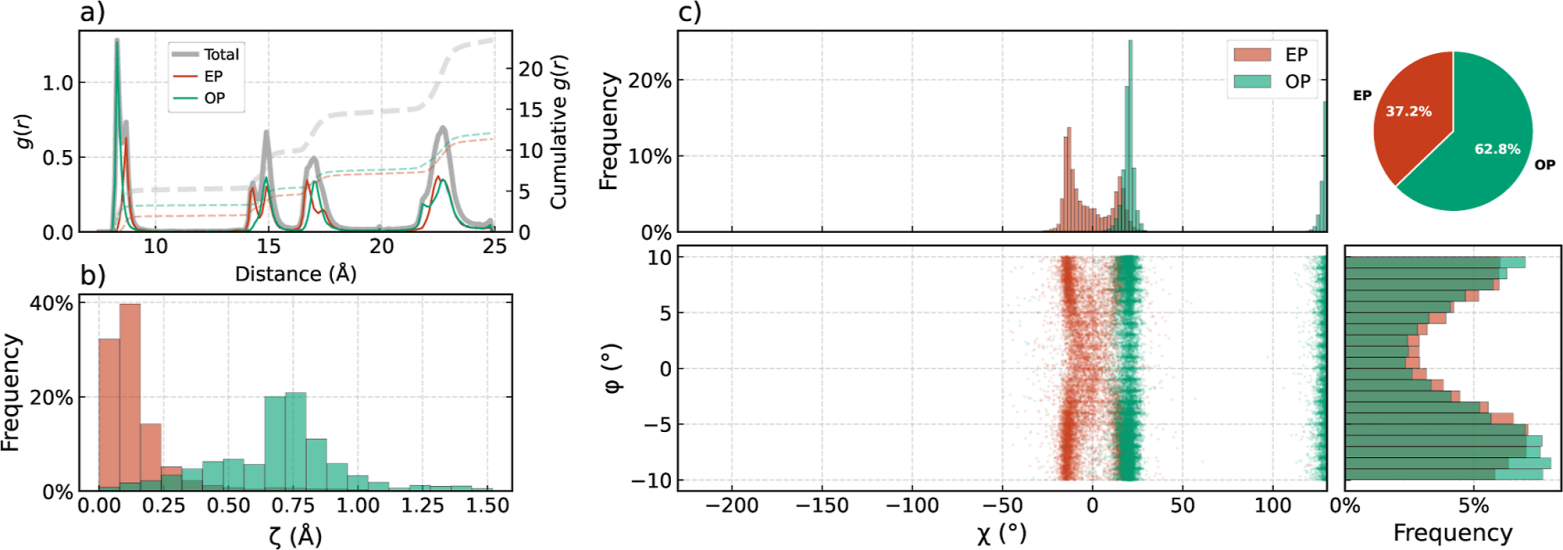
Structural analysis of the {L↑-D↑} system. (a) Radial distribution function (RDF) showing the two-peak feature indicating different EP and OP neighbor distances. (b) Distribution of the relative offset ζ for EP interactions. (c) Joint distribution of the angle difference χ and relative axial offset ζ for EP interactions. The loss of a well-defined optimal orientation for EP pairs indicates frustration induced by the preferred, incompatible OP configuration.

Furthermore, RONN features are considered by analyzing statistical distributions of ζ, φ_1_, and χ. Figure 9b shows that the sharp distribution at ζ = 0 Å for EP interactions is lost, replaced by a broad peak with maximum near 0.1 Å. Additionally, as indicated by the scatter plot in Figure 9c, the defined distributions in φ_1_ and χ are also lost. Instead, the distribution in φ_1_ ranges over the entire interval from −10° to 10° with a maximum near −8°, and two dominant values for χ near −20° and 20° are observed.

For OP interactions, the distribution of relative offset ζ ranges over the entire interval with a dominant peak at approximately ±0.75 Å. Similar to EP interactions, the distribution of φ_1_ spreads over the full range from −10° to 10° but exhibits a less well-defined peak near −7°, while two rather sharp peaks are obtained for χ near 20° and 130°.

Comparing the obtained χ values for OP interactions with the pair interaction potential presented in Section 3.1 reveals that the system drives toward optimal OP arrangement. However, since this configuration is incompatible with the hexagonal lattice, frustration affects the remaining interactions, particularly EP interactions, as the interaction energy of EP configuration is approximately 0.4 eV smaller than that of the OP configuration.

Turning to the {L↑↓-D↑↓} system (racemic mixture with mixed up and down alignments, Figure 7d), both effects reported so far appear to occur. On one hand, formation of parallel lines of alternating handedness is observed. On the other hand, as indicated by the highlighted regions in Figure 7d, domains exist where right-handed helices are upward-aligned while left-handed ones are oriented downward, or vice versa. Preferential pairing between helices of opposite handedness and direction can be concluded from pair interaction potentials, which showed the overall most stable configuration for OA orientation. Consequently, the tendency to adopt this configuration governs the self-assembled film properties, driving the system toward the described features.

To better understand which orientations are preferentially adopted, we considered the {L↑-D↓} system (where all right-handed helices are oriented upward and left-handed ones downward), resulting in the structure exemplarily shown in Figure 7e. Figure 10 displays the obtained characteristics for RDF and RONN. The complete statistical analysis of the {L↑↓-D↑↓} system is presented in detail in the Supporting Information, section S4.2, where in Figure S9 the obtained RDF and RONN distribution are shown.

**Figure 10:**
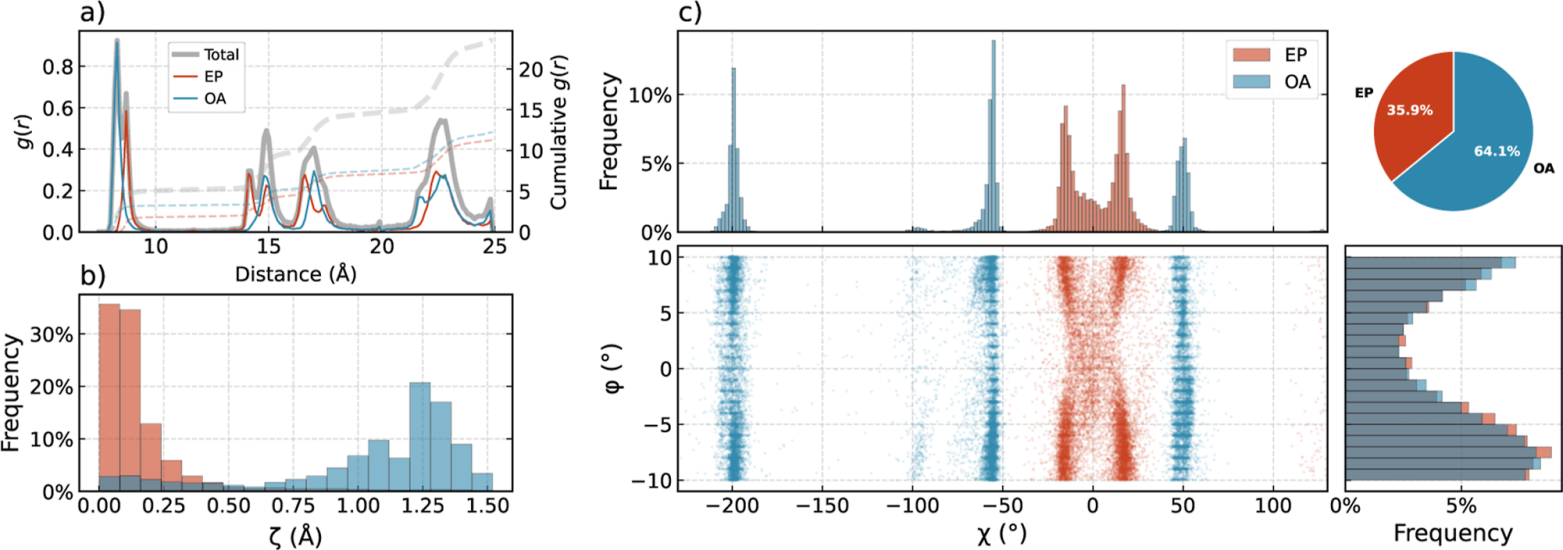
Structural analysis of the {L↑-D↓} system. (a) Radial distribution function (RDF). (b) Distribution of the angle φ_1_ for EP interactions. (c) Joint distribution of the angle difference χ and relative axial offset ζ for EP interactions. The OA interactions show a distinct signature, confirming the drive to form optimal opposite-handed, antiparallel pairs.

Again, the hexagonal lattice is slightly deformed with smaller distances between helices of opposite handedness due to energetically favored OA interaction, see Figure 10a. As indicated by Figure 10b and c, the EP interaction statistics reveal the same features as discussed for the {L↑-D↑} case (Figure 9): a broadened ζ distribution with maximum at 0 Å, φ_1_ ranging over the full interval with maximum near −8°, and two pronounced peaks near χ = −10° and χ = 10°. The OA interaction distributions, however, differ significantly. While φ_1_ follows the EP trend with a broad distribution peaking at −8°, the χ distribution indicates three distinct peaks, while the ζ distribution spreads over the entire range with the largest contribution near −0.3 Å.

Comparing these configurations with low-energy configurations presented in Section 3.2 reveals that self-assembled structures successfully achieve a high proportion of optimal OA pairings. However, geometric constraints of the hexagonal lattice introduce frustration, preventing all pairs from reaching the absolute global minimum configuration and resulting in the observed distributions for both EP and OA interaction parameters. In conclusion, we find that the theoretical model predicts that racemic mixtures of right- and left-handed αPA self-assemble into parallel rows of alternating handedness, giving rise to a rectangular phase. This result confirms the interpretation drawn from STM measurements in which the rectangular dimer phase characterized by stripe-like features was assumed to be formed by helices of opposite handedness. The two-peak distribution in χ for EP interaction (see Figures 9c and 10c) suggests the formation of two different angular arrangements. Assuming that the maximum features measured in STM experiments are not centered over the helix, this angular difference manifests as different distances, which are indeed observed experimentally (Figure 2b, red line).

A direct structural interpretation of the apparent height displacement by approximately 2.5 Å within the dimer concluded from STM images is not straightforward if one assumes parallel-aligned helices with the same termination groups at the surface. It would imply either that helices are completely offset by about two alanine units (leaving a gap at the SAM-substrate interface), as proposed earlier, or that helices are stretched. Such an offset would, however, decrease the overall interaction region, reducing the energy gain from alignment by up to 150 meV per interaction. A helix stretching, on the other hand, is also unlikely since this would break the intrahelix H-bonds, accounting for approximately 0.4 eV per broken H-bond.^30^ Both scenarios are therefore unlikely to explain the observed offset.

As our theoretical study now suggests, opposite-handed helices adopt an antiparallel orientation, which enables formation of the most stable and densely packed configuration identified from the OA interaction potential. Guided by these simulation results, a more plausible explanation is that the apparent height modulation originates from the antiparallel arrangement of helices bearing chemically distinct terminal groups. The changed interfacial interactions resulting from the up/down orientation affecting both the helix-tip and the helix-substrate interfaces give rise to contrast variations in STM images. We note that the different termination groups may lead to a slightly modified helix-substrate separation, but such an actual height difference cannot account for the experimentally observed 2.5 Å of apparent height modulation, for the same reasons discussed for the parallel-aligned case. This interpretation reconciles the theoretical prediction of antiparallel, opposite-handed dimer rows with the experimentally observed stripe-like patterns and provides a unified picture of molecular ordering within self-assembled α-polyalanine monolayers.

## 4 Conclusion

In this study, we have developed a theoretical framework based on an effective potential derived from SCC-DFTB calculations to investigate the self-assembly of α-polyalanine (αPA) at the molecular scale. We analyzed the generated interaction potentials and identified the specific intermolecular interactions that stabilize particular helix arrangements in isolated dimers. Our systematic investigation revealed that relative handedness and axial orientation of adjacent helices are the primary determinants of interhelical packing. We quantitatively demonstrated that opposite-handed helices in antiparallel (OA) and parallel (OP) alignments exhibit significantly stronger binding energies and closer equilibrium distances than their equal-handed counterparts (EP, EA). This fundamental energetic preference, rooted in superior interdigitation, provides a direct atomistic rationale for Wallach’s rule, explaining the denser packing observed in racemic mixtures.

By employing the generated effective potentials in heuristic optimization using simulated annealing, we successfully predicted self-assembled monolayer structures that replicate key structural motifs observed in experimental STM measurements. For enantiopure systems {L↑}, the dominance of EP interactions naturally leads to frustration-free, hexagonally close-packed (hcp) lattices where every neighbor pair adopts the optimal configuration. In contrast, racemic mixtures are driven by the superior stability of opposite-handed interactions, particularly in antiparallel alignment, which induces structural frustration within the hexagonal lattice. This drives the formation of stripe-like phases with alternating chirality and rectangular structures, in excellent agreement with STM observations.

Crucially, our simulations provide a novel and more plausible interpretation of the apparent height modulation in STM images of the racemic dimer phase. Based on our theoretical results, we conclude that the contrast does not arise from substantial physical offset or stretching of parallel helices—scenarios that are energetically unfavorable—but rather from the antiparallel alignment of opposite-handed helices bearing chemically distinct terminal groups. The differing electronic properties of these end groups at the substrate interface would naturally produce the observed height contrast in constant-current STM, reconciling experimental data with our theoretical prediction of antiparallel, OA-stabilized dimer rows.

These findings establish robust structure–property relationships for polypeptide SAMs, directly linking chiral composition and molecular orientation to supramolecular order. The identified interaction motifs are critical not only for structural stability but also have profound implications for electronic and spintronic properties. The precise control over dipole orientation and intermolecular coupling, dictated by the identified low-energy configurations, represents a key factor modulating the Chiral-Induced Spin Selectivity (CISS) effect, explaining variations in magnetoresistance between different SAM phases.

In summary, this work moves beyond a phenomenological description to provide a predictive molecular model for chiral peptide self-assembly. The insights gained form a solid foundation for the rational design of peptide-based materials with tailored supramolecular order. Future work will integrate these structural models with charge transport calculations to explicitly unravel the mechanism of spin-selective conduction in these complex, yet elegantly ordered, biomolecular systems.

The methodological approach presented here—systematically parametrizing interactions of isolated helical dimers to construct effective potentials for SAM films—is broadly applicable and can be extended to other polypeptide systems. Of particular interest are peptides adopting non-α-helical structures. In such systems, geometric frustration effects may emerge even in enantiopure monolayers, potentially leading to novel supramolecular architectures beyond the hexagonal and rectangular phases observed for αPA. Furthermore, the generated effective potentials can be employed in kinetic Monte Carlo or molecular dynamics simulations to probe nonequilibrium kinetics of SAM formation, providing insights into nucleation, growth kinetics, and domain boundary formation.

Finally, the SCC-DFTB framework provides not only structural parameters but also complete electronic structure information for isolated helices and their pairs. This electronic foundation enables subsequent investigations into the origin of the Chiral-Induced Spin Selectivity effect, allowing direct mapping between specific molecular configurations and their spin-filtering capabilities, thereby bridging the gap between supramolecular organization and spintronic functionality. Future refinements may incorporate higher-level DFTB variants or first-principles benchmarks, although such changes are not expected to alter the qualitative conclusions of this work.

## Supplementary Material



## Notes

S1. Structure formation during Simulated annealing, S2. Illustrative Flowchart, S3. Helix-Pair Interactions of EA and OP alignments, S4. Statistical Analysis of Low-Energy Configurations of Self-Assembled {L↑↓} and {L↑↓-D↑↓} Films (PDF)

## References

[1] Mrksich M. (2009). Using self-assembled
monolayers to model the extracellular matrix. Acta Biomater..

[2] Gatto E., Venanzi M. (2013). Self-assembled monolayers formed
by helical peptide
building blocks: a new tool for bioinspired nanotechnology. Polym. J..

[3] Banerjee-Ghosh K., Ben Dor O., Tassinari F., Capua E., Yochelis S., Capua A., Yang S.-H., Parkin S. S. P., Sarkar S., Kronik L., Baczewski L. T., Naaman R., Paltiel Y. (2018). Separation
of enantiomers by their enantiospecific interaction with achiral magnetic
substrates. Science.

[4] Bloom B. P., Paltiel Y., Naaman R., Waldeck D. H. (2024). Chiral Induced Spin
Selectivity. Chem. Rev..

[5] Soumyanarayanan A., Reyren N., Fert A., Panagopoulos C. (2016). Emergent phenomena
induced by spin–orbit coupling at surfaces and interfaces. Nature.

[6] Dong Y., Hautzinger M. P., Haque M. A., Beard M. C. (2025). Chirality-Induced
Spin Selectivity in Hybrid Organic-Inorganic Perovskite Semiconductors. Annual Review of Physical Chemistry.

[7] Sharma A., Matthes P., Soldatov I., Arekapudi S. S. P. K., Bohm B., Lindner M., Selyshchev O., Thi Ngoc Ha N., Mehring M., Tegenkamp C., Schulz S. E, Zahn D. R. T., Paltiel Y., Hellwig O., Salvan G. (2020). Control of magneto-optical properties of cobalt-layers
by adsorption of α-helical polyalanine self-assembled monolayers. J. Mater. Chem. C.

[8] Sek S., Tolak A., Misicka A., Palys B., Bilewicz R. (2005). Asymmetry
of Electron Transmission through Monolayers of Helical Polyalanine
Adsorbed on Gold Surfaces. J. Phys. Chem. B.

[9] Blondelle S. E., Forood B., Houghten R. A., Pérez-Payá E. (1997). Polyalanine-Based
Peptides as Models for Self-Associated β-Pleated-Sheet Complexes. Biochemistry.

[10] Ghosh S., Mishra S., Avigad E., Bloom B. P., Baczewski L. T., Yochelis S., Paltiel Y., Naaman R., Waldeck D. H. (2020). Effect
of Chiral Molecules on the Electron’s Spin Wavefunction at
Interfaces. J. Phys. Chem. Lett..

[11] Moharana A., Kapon Y., Kammerbauer F., Anthofer D., Yochelis S., Shema H., Gross E., Klaui M., Paltiel Y., Wittmann A. (2025). Chiral-induced unidirectional
spin-to-charge conversion. Sci. Adv..

[12] Chen S., Wu R., Fu H.-H. (2024). Persistent
Chirality-Induced Spin-Selectivity Effect
in Circular Helix Molecules. Nano Lett..

[13] Nguyen T. N. H., Solonenko D., Selyshchev O., Vogt P., Zahn D. R. T., Yochelis S., Paltiel Y., Tegenkamp C. (2019). Helical Ordering
of α-l-Polyalanine Molecular Layers by Interdigitation. J. Phys. Chem. C.

[14] Nguyen T. N. H., Xue S., Tegenkamp C. (2020). Heterochiral
Dimer Formation of α-l-
and α-d-Polyalanine Molecules on Surfaces. J. Phys. Chem. C.

[15] Subotnik J. E. (2023). Chiral
molecules to transmit electron spin. Science.

[16] Safari M. R., Matthes F., Schneider C. M., Ernst K.-H., Burgler D. E. (2024). Spin-Selective
Electron Transport Through Single Chiral Molecules. Small.

[17] Chiesa A., Privitera A., Garlatti E., Allodi G., Bittl R., Wasielewski M. R., Sessoli R., Carretta S. (2025). Chirality-Induced Spin
Selectivity at the Molecular Level: A Different Perspective to Understand
and Exploit the Phenomenon. J. Phys. Chem. Lett..

[18] Kohtani M., Jarrold M. F. (2004). Water Molecule Adsorption
on Short Alanine Peptides: 
How Short Is the Shortest Gas-Phase Alanine-Based Helix?. J. Am. Chem. Soc..

[19] Hoffmann W., Marianski M., Warnke S., Seo J., Baldauf C., von Helden G., Pagel K. (2016). Assessing the stability of alanine-based
helices by conformer-selective IR spectroscopy. Phys. Chem. Chem. Phys..

[20] O’Neil K. T., DeGrado W. F. (1990). A Thermodynamic Scale for the Helix-Forming Tendencies
of the Commonly Occurring Amino Acids. Science.

[21] Ha
Nguyen T. N., Paltiel Y., Baczewski L. T., Tegenkamp C. (2023). Spin Polarization of Polyalanine Molecules in 2D and
Dimer-Row Assemblies Adsorbed on Magnetic Substrates: The Role of
Coupling, Chirality, and Coordination. ACS Appl.
Mater. Interfaces.

[22] Wallach O. (1895). Zur Kenntniss
der Terpene und der ätherischen Oele. Justus Liebigs Ann. Chem..

[23] Ha N. T. N., Sharma A., Slawig D., Yochelis S., Paltiel Y., Zahn D. R. T., Salvan G., Tegenkamp C. (2020). Charge-Ordered
α-Helical Polypeptide Monolayers on Au(111). J. Phys. Chem. C.

[24] Nguyen T. N. H., Salvan G., Hellwig O., Paltiel Y., Baczewski L. T., Tegenkamp C. (2024). The mechanism of the molecular CISS
effect in chiral
nano-junctions. Chem. Sci..

[25] Elstner M., Porezag D., Jungnickel G., Elsner J., Haugk M., Frauenheim T., Suhai S., Seifert G. (1998). Self-consistent-charge
density-functional tight-binding method for simulations of complex
materials properties. Phys. Rev. B.

[26] Hourahine B. (2020). DFTB+, a software package
for efficient approximate density functional
theory based atomistic simulations. Chem. Phys..

[27] Rappé A. K., Casewit C. J., Colwell K. S., Goddard W. A., Skiff W. M. (1992). UFF, a
full periodic table force field for molecular mechanics and molecular
dynamics simulations. J. Am. Chem. Soc..

[28] Christensen A. S., Kubar T., Cui Q., Elstner M. (2016). Semiempirical Quantum
Mechanical Methods for Noncovalent Interactions for Chemical and Biochemical
Applications. Chem. Rev..

[29] Dutta J., Sahu A. K., Jena S., Biswal H. S. (2025). Methyl Interactions
in Proteins: Insights from Structural and Computational Studies. J. Chem. Inf. Model..

[30] Lantz M. A., Jarvis S. P., Tokumoto H., Martynski T., Kusumi T., Nakamura C., Miyake J. (1999). Stretching the α-helix:
a direct measure of the hydrogen-bond energy of a single-peptide molecule. Chem. Phys. Lett..

