# Supplementary material for: Simulation of Self-Assembled Monolayers of Polyalanine α‑Helices: Development and Application of an Effective Potential for Film Structure Predictions
Source: ACS Appl Mater Interfaces. 2026 May 22;18(21):30467–79. doi: 10.1021/acsami.6c01087 (PMC13244372; doi:10.1021/acsami.6c01087)
Supplement: Supplementary file 1 [file am6c01087_si_001.pdf]

Supporting Information

Simulation of Self-Assembled Monolayers of Polyalanine α-Helices: Development and Application of an Effective Potential for Film Structure Predictions

Hadis Ghodrati[1], Kevin Preis[1], Thi Ngoc Ha Nguyen[1], Christoph Tegenkamp[1], Sibylle Gemming[1], Jeffrey Kelling[1,2], Florian Günther[3,*]

1. Institute of Physics, Technische Universität Chemnitz, 09107 Chemnitz, Germany
2. Institute for Radiation Physics, Helmholtz-Zentrum Dresden - Rossendorf, 01328 Dresden, Germany
3. Departamento de Física, Universidade Estadual Paulista, Instituto de Geociências e Ciências Exatas, 13506-900 Rio Claro, SP, Brazil

*Email: florian.gunther@unesp.br

S1 Structure Formation during Simulated Annealing

In Figure S1, the heat capacity $C_\mathrm{V}(T)$ as a function of temperature T is shown for the different ensembles. During the MC simulations, the heat capacity was evaluated from fluctuations of the internal energy. For a canonical ensemble, it follows from the fluctuation-dissipation theorem:

$$C_\mathrm{V}(T) = \left(\frac{\partial U}{\partial T}\right)_{V=\mathrm{const}} = \frac{\langle E^2\rangle - \langle E\rangle^2}{k_\mathrm{B}T^2}\,, \tag{1}$$

where U is the internal energy of the system. The ensemble averages $\langle E\rangle$ and $\langle E^2\rangle$ are obtained from the Markov chain by simple averaging,

$$\langle F\rangle \approx \frac{1}{N}\sum_{i=0}^{N-1} F(\vec{x}_i)\,, \tag{2}$$

with $\vec{x}_i$ denoting the generated microstates.

For simulation temperatures $T > 10000\,\mathrm{K}$, all systems are in an amorphous state, meaning that thermal fluctuations dominate over the attractive interactions between the helices. The enantiomerically pure systems show a sharp peak in the heat capacity at approximately $T = 2800\,\mathrm{K}$, whereas the racemic systems display a similar peak at a slightly lower temperature of about $T = 2500\,\mathrm{K}$. These pronounced peaks identify the temperature regions where regular structures emerge and the packing density increases, indicating that attractive interactions start to outweigh thermal motion.

Around 500 K, the $\{L\uparrow\downarrow\}$ systems exhibit a small additional local maximum in their heat capacity that is absent in the other systems. At this simulation temperature, demixing into enantiopure domains occurs. At simulation temperatures below 500 K, all systems first show a decrease in heat capacity before another small local maximum appears between roughly 1 K and 20 K. For even lower temperatures, the heat capacity steadily approaches $0\,\mathrm{eVK^{-1}}$, with this decline occurring much faster in the racemates. In these systems, the increased frustration suppresses changes in the degrees of freedom of individual helices, making energy-lowering rearrangements increasingly improbable.

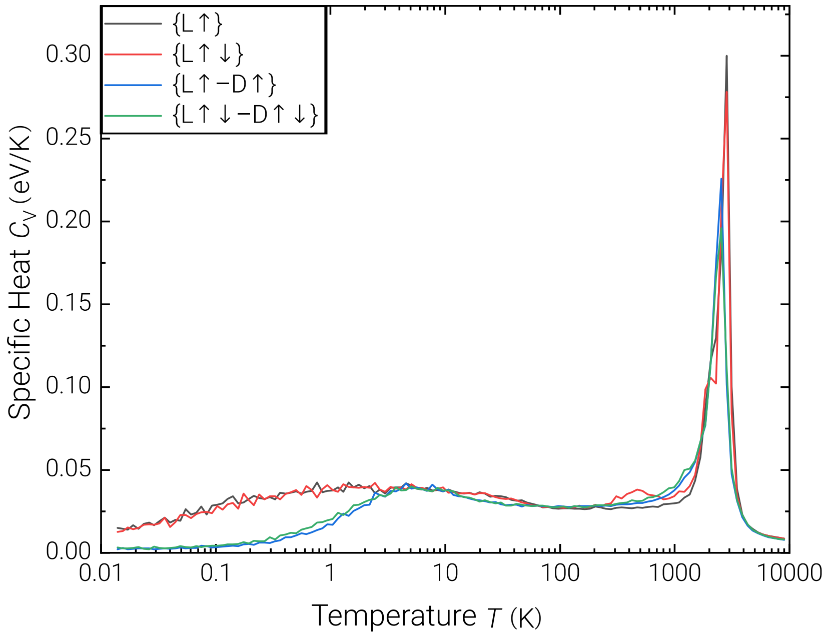

Figure S1: Heat capacity $C_\mathrm{V}(T)$ for the different PA systems.

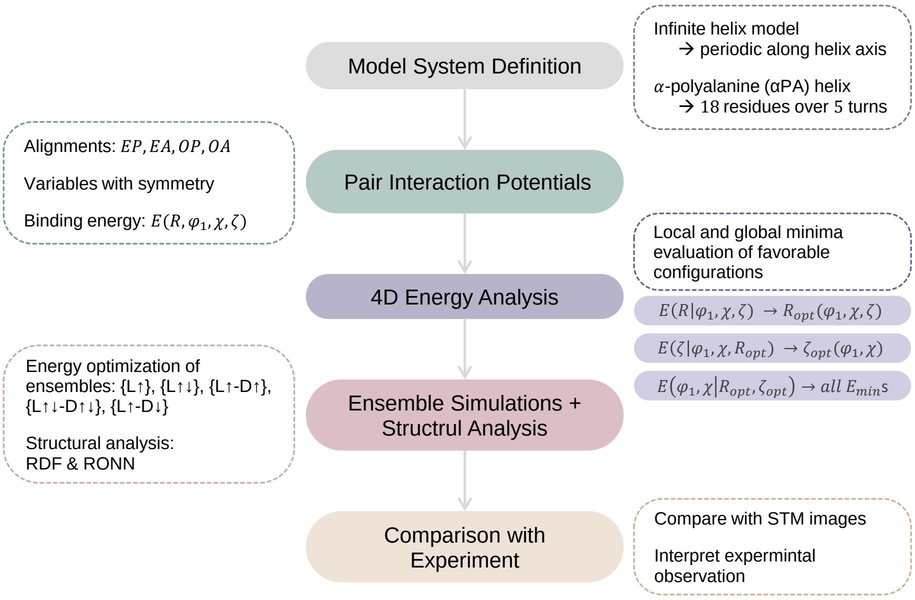

Figure S2: Flowchart illustrating the stepwise theoretical approach: (1) DFTB calculations of isolated helices; (2) systematic sampling of inter-helical parameters to compute pair interaction energies; (3) extraction of effective potentials for EP, EA, OP, and OA configurations; (4) simulated annealing of enantiopure and racemic ensembles; (5) statistical analysis of structural properties (radial distribution functions, nearest-neighbor orientations) and comparison with experimental STM data.

S2 Illustrative Flowchart

To provide a clear overview of the theoretical methodology, a flowchart summarizing the sequential steps of our study is presented in Fig. S2. The workflow begins with *ab initio* DFTB calculations of isolated α-PA helices to establish reference energies. Subsequently, a systematic sampling of the four-dimensional parameter space (R, φ_1, χ, ζ) for all four pair configurations (EP, EA, OP, OA) is performed. From these data, effective pair potentials are extracted and used in simulated annealing simulations of molecular ensembles (160 helices) to predict low-energy self-assembled structures. Finally, structural features such as radial distribution functions and nearest-neighbor orientations are statistically analyzed to compare with experimental STM observations.

S3 Helix-Pair Interactions of EA and OP Alignments

S3.1 Distance-Dependent Interaction Energy

The binding energy curves and optimal distances for all simulated configurations discussed in Section 3.1 of the main text are presented here for the EA and OP cases. Figure S3a illustrates binding energy $E_{\text{bind}}(R)$ curves for four selected relative orientations in EA alignment (colored lines), with the corresponding (φ_1,χ,ζ) values indicated in the legend. Figure S3b presents corresponding results for OP alignment, where helices possess opposite handedness and are oriented parallel. The analysis reveals systematic trends in binding strength and equilibrium spacing across different orientation combinations.

To provide a comprehensive comparison of different pair interactions, Figure S4 overlays the boundary distributions of optimal distances for all possible pair configurations across the four interaction types. This overview highlights the systematic differences in packing preferences between same-handed and opposite-handed helix pairs.

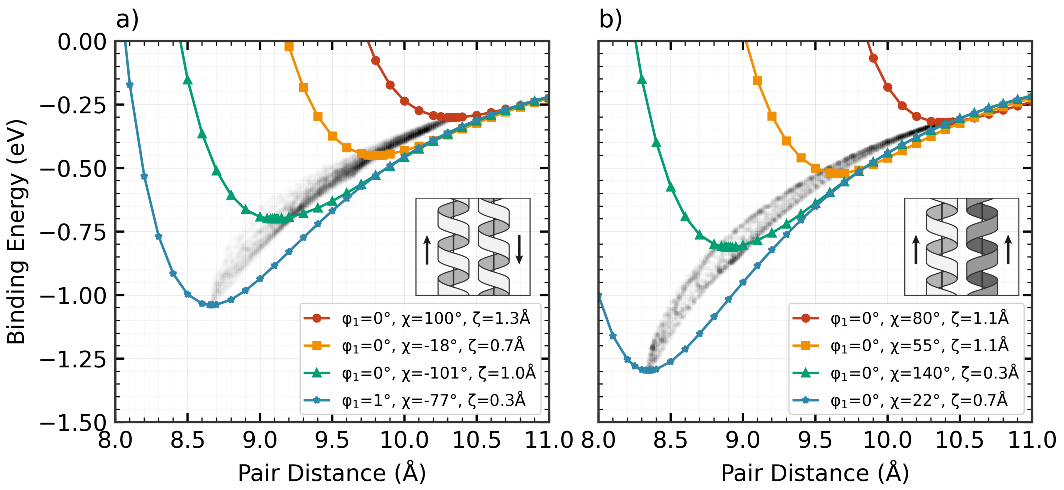

Figure S3: Distance-dependent binding energy profiles for (a) EA (equal-handed, anti-parallel) and (b) OP (opposite-handed, parallel) configurations. The curves demonstrate the variation in binding strength and optimal separation distance across different relative orientations.

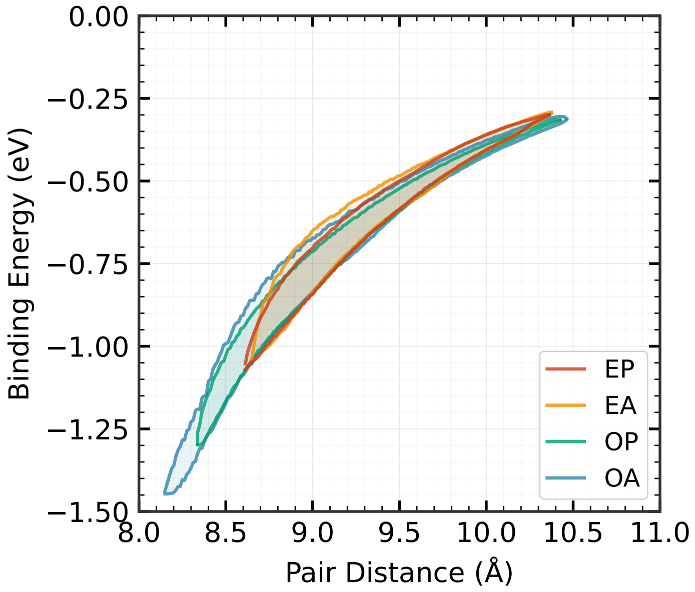

Figure S4: Distribution of optimal distances for all configurations across the four interaction types (EP, EA, OP, OA).

S3.2 Dependence on Relative Offset

In the main text, we investigated how the binding energy depends on the height difference and pair distances for parallel equal-handed (EP) and anti-parallel opposite-handed (OA) configurations. Figure S5 extends this analysis to the EA and OP cases, showing how the relative vertical offset ζ influences the optimal inter-helical distance and binding energy for these interaction types.

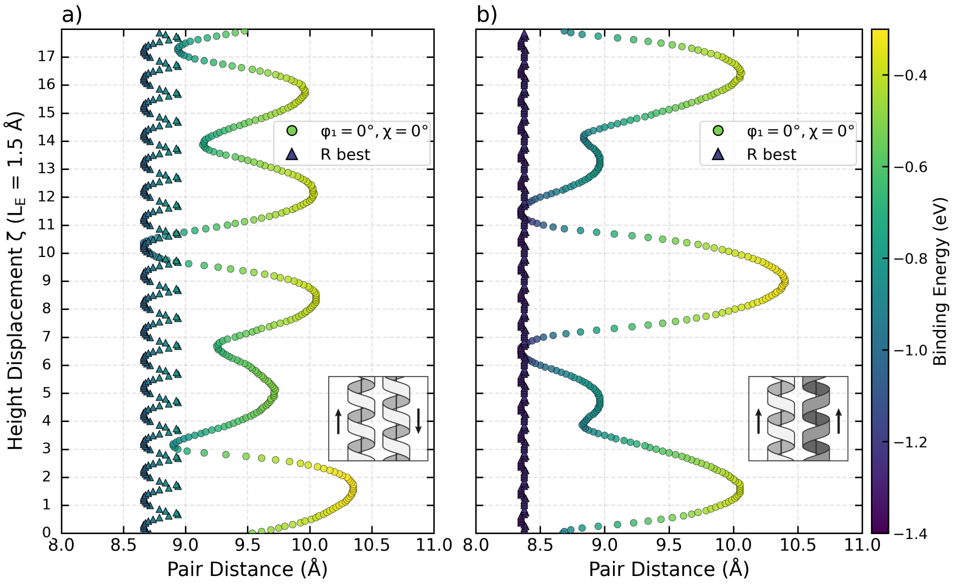

Figure S5: Optimal pair distance and binding energy as a function of relative vertical offset ζ for (a) EA and (b) OP configurations. The oscillatory patterns reflect the periodic nature of helical interactions and the influence of side-chain interdigitation.

S3.3 Angle Dependence

While the main text discussed the angular dependence for equal-handed parallel (EP) and opposite-handed anti-parallel (OA) pairs in detail, we now examine the remaining cases. Figure S6 shows the angular dependence for equal-handed anti-parallel (EA) and opposite-handed parallel (OP) configurations. For EA pairs, the binding-energy landscape exhibits minima around $\varphi_1 = 0°$ and $\chi \approx -70°$. The EA configuration shows a more complex angular dependence, whereas OP exhibits stripe-like features similar to OA interactions.

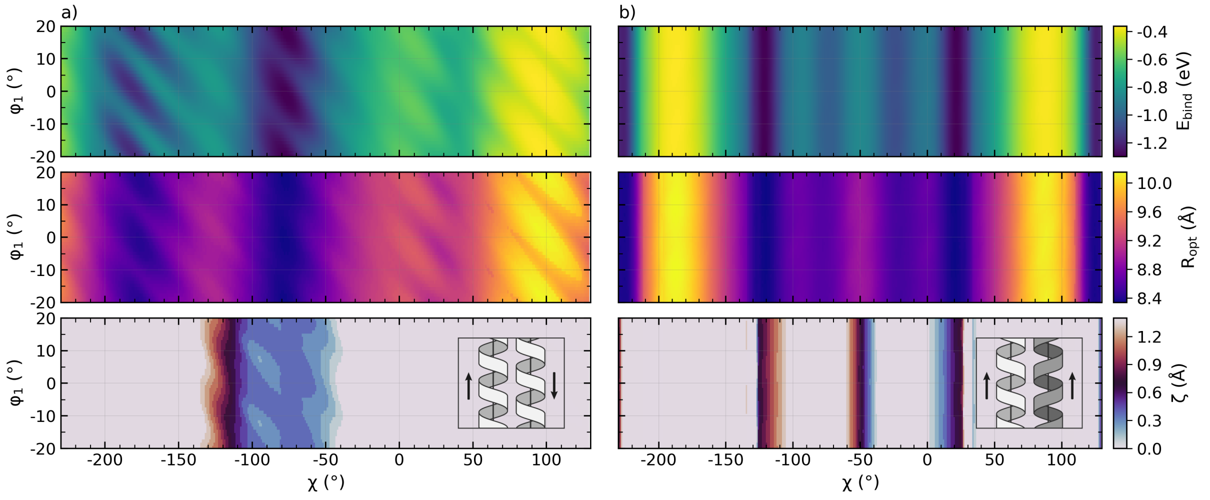

Figure S6: Heatmaps of binding energy, equilibrium distance R_{opt}, and relative offset ζ_{opt} as functions of angular parameters φ_1 and χ for (a) EA and (b) OP configurations.

S3.3.1 Local Interactions in Minimum-Energy Configurations

The global minimum configurations for EA and OP helix pairs reveal distinct structural motifs that complement the EP and OA structures discussed in the main text. For EA alignment, the optimal configuration occurs at specific angular parameters that maximize interdigitation while maintaining

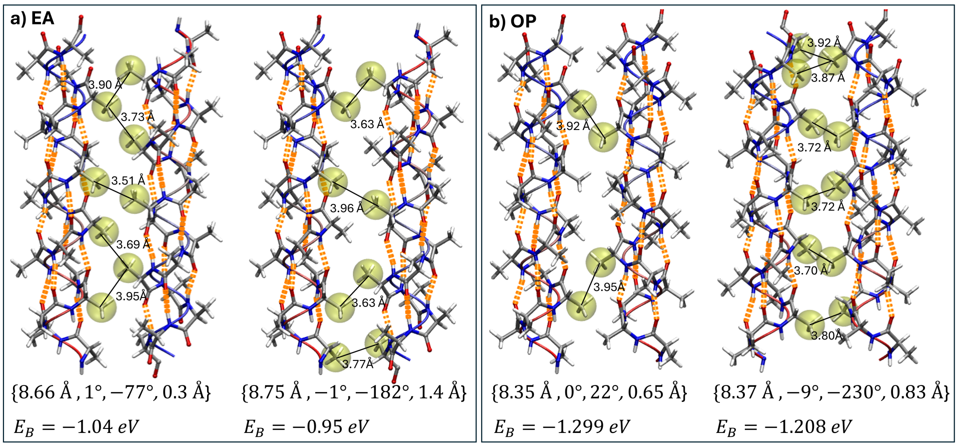

Figure S7: Representative low-energy configurations of EA (a) and OP (b) helix pairs. For better visualization, the helical backbone is represented as a coil. The specific relative configuration parameters R, φ_1, χ, ζ, as well as the corresponding binding energies E_B, are given below each configuration. Intramolecular hydrogen bonds stabilizing the α helix are indicated in orange. Methyl groups with inter-helical distances smaller than 4 Å are highlighted by yellow spheres, with the individual distances given in the figure.

anti-parallel orientation. The OP global minimum configuration demonstrates how opposite-handed helices in parallel alignment achieve close packing through complementary side-chain arrangements. These structures provide insight into the diverse packing strategies available to helical polypeptides.

S4 Statistical Analysis of Low-Energy Configurations of Self-Assembled Films

S4.1 Structural Properties of Mixed Parallel and Antiparallel Enantiopure Films

As shown in Figure S8, the {L↑↓} system exhibits complex domain formation with both EP and EA interactions coexisting within the same film. The radial distribution function shows characteristic features of domain boundaries, while the RONN analysis reveals how helices adapt their orientations at these interfaces. The statistical distributions provide quantitative insight into the structural frustration induced by mixed molecular orientations.

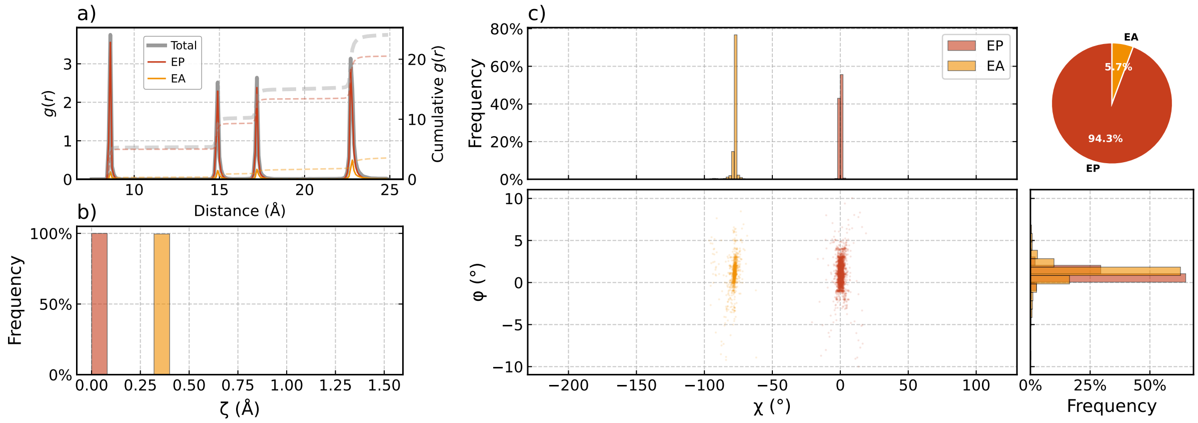

Figure S8: Structural analysis of the {L↑↓} system showing (a) radial distribution function with domain boundary signatures, (b) distribution of relative offset ζ for EA interactions, and (c) joint distribution of angular parameters for nearest neighbors at domain boundaries.

S4.2 Structural Properties of Mixed Parallel and Anti-Parallel Racemic Films

The fully mixed racemic system {L↑↓-D↑↓} represents the most complex scenario, with all four interaction types present (see Figure S9). The structural analysis reveals how the competing preferences for OA and OP interactions lead to packing arrangements that differ from both enantiopure and simpler racemic systems. The RDF and RONN statistics provide evidence for the formation of distinct structural domains driven by specific interaction preferences.

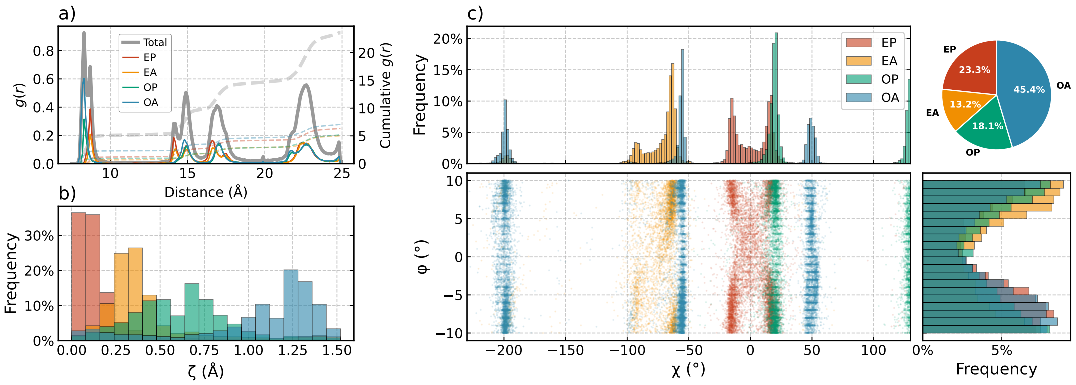

Figure S9: Comprehensive structural analysis of the {L↑↓-D↑↓} system, including (a) radial distribution function showing complex peak structure, (b and c) RONN statistics for the different interaction types.
